# Establishment of a 7-gene prognostic signature based on oxidative stress genes for predicting chemotherapy resistance in pancreatic cancer

**DOI:** 10.3389/fphar.2023.1091378

**Published:** 2023-04-17

**Authors:** Shengmin Zhang, Jianrong Yang, Hongsheng Wu, Tiansheng Cao, Tengfei Ji

**Affiliations:** Department of Hepatobiliary Surgery, Affiliated Huadu Hospital, Huadu People’s Hospital, Guangzhou, Guangdong, China

**Keywords:** pancreatic cancer, oxidative stress, methylation, molecular subtypes, risk score, small molecule chemotherapeutic drugs, prognosis, tumor immunity

## Abstract

**Background:** Oxidative stress is involved in regulating various biological processes in human cancers. However, the effect of oxidative stress on pancreatic adenocarcinoma (PAAD) remained unclear.

**Methods:** Pancreatic cancer expression profiles from TCGA were downloaded. Consensus ClusterPlus helped classify molecular subtypes based on PAAD prognosis-associated oxidative stress genes. Limma package filtered differentially expressed genes (DEGs) between subtypes. A multi-gene risk model was developed using Lease absolute shrinkage and selection operator (Lasso)-Cox analysis. A nomogram was built based on risk score and distinct clinical features.

**Results:** Consistent clustering identified 3 stable molecular subtypes (C1, C2, C3) based on oxidative stress-associated genes. Particularly, C3 had the optimal prognosis with the greatest mutation frequency, activate cell cycle pathway in an immunosuppressed status. Lasso and univariate cox regression analysis selected 7 oxidative stress phenotype-associated key genes, based on which we constructed a robust prognostic risk model independent of clinicopathological features with stable predictive performance in independent datasets. High-risk group was found to be more sensitive to small molecule chemotherapeutic drugs including Gemcitabine, Cisplatin, Erlotinib and Dasatinib. The 6 of 7 genes expressions were significantly associated with methylation. Survival prediction and prognostic model was further improved through a decision tree model by combining clinicopathological features with RiskScore.

**Conclusion:** The risk model containing seven oxidative stress-related genes may have a greater potential to assist clinical treatment decision-making and prognosis determination.

## Introduction

Pancreatic adenocarcinoma (PAAD) is one of the most difficult malignancies to treat (Katona et al., 2021), with gallstones, chronic pancreatitis, smoking, alcohol drinking as the most common risk factors for PAAD (Lowenfels et al., 1993). Ductal adenocarcinoma of the pancreas is the predominant histopathological type accounting for 85% of all the PAAD cases. Surgical resection is not available to proximately 80%–85% of patients due to a lack of typical manifestations at the initial stage (Singhi et al., 2019). For those patients with PAAD who have taken surgery, 5-year overall survival probability is only about 20% (Wu et al., 2019). The technology of genome sequencing has further characterized the molecular patterns and genotypic heterogeneity of pancreatic cancer. Given that molecular targeting therapies have become indispensable in treatment, discovering new therapeutic targets is crucial. Hence, for improving the prognostic prediction of PAAD, it is imperative to identify novel prognostic indicators.

Oxidative stress functions importantly in pathogenesis of multiple diseases, including inflammatory diseases, cancer, and immune-mediated (Azmanova and Pitto-Barry, 2022). Oxidative stress induces reactive oxygen species (ROS) that could damage lipids, proteins, DNA, and produce mutagenic metabolites to affect tumor biological behaviors and transform malignant phenotype (Sosa et al., 2013). Tumor microenvironment consists of surrounding tissue components and interacting tumor cells, with the latter favoring biological behaviors of tumor cells. ROS has a complex and multifaceted role in tumor microenvironment. A study found that the non-classical glutamine pathway promotes the development of pancreatic cancer with dysregulation of oxidative stress (Son et al., 2013). ROS inhibits the arginine methylation enzyme CARM1, which in turn inhibits MDH1 activity. Thus, ROS could activate non-classical glutamine metabolism to promote pancreatic cancer cell growth (Son et al., 2013). Glutamine and asparagine are two key nutrients affecting pancreatic cancer cell development, moreover, these two are one of the bases of protein synthesis in pancreatic cancer cells to promote resistance to oxidative stress and are essential for pancreatic cancer cell growth and proliferation. Pathria et al. showed that simultaneous inhibition of asparagine metabolic pathway and MAPK pathway inhibited pancreatic cancer development (Pathria et al., 2019). Methionine residues has been found to serve as a reversible redox switch in controlling different signaling outcomes. To control tumor metastasis, MSRA-PKM2 axis is a regulatory bridge between cancer metabolism and redox biology (He et al., 2022). Therefore, future studies on the role of oxidative stress in PAAD and the impact on TME are needed to optimize immunotherapy or develop new therapeutic strategies.

Consistent clustering screened stable molecular subtypes utilizing genes of oxidative stress pathway. We also compared immune features, mutational, clinical pathway features among the subtypes. Finally, we identified genes associated with oxidative stress phenotypes using differential expression analysis and LASSO. Moreover, a risk model and clinical prognostic model was developed for facilitating personalized PAAD treatment.

## Materials and methods

### Data collection and processing

We used TCGA GDC API to download the mutation data and RNA-seq data [transcripts per million (TPM)] of TCGA-PAAD. A total of 176 primary tumor samples were finally obtained after screening. We downloaded transcriptomic data of samples from the pancreatic cancer-Australia (PACA-AU) and pancreatic cancer-Canada (PACA-CA) cohorts in the International Cancer Genome Consortium (ICGC) database (https://dcc.icgc.org/projects), with each cohort containing 267 and 215 pancreatic cancer samples, respectively. Oxidative stress-related genes were obtained e oxidative stress pathway “GOBP_RESPONSE_TO_OXIDATIVE_STRESS” in MSigDB database.

### Data pre-processing

The RNA-seq data from TCGA were preprocessed as follows.

1) Removing samples that did not contain clinical information of follow-up;
2) Removing samples that did not show survival time;
3) Removing samples that did not show status;
4) Conversion of Ensembl to Gene symbol;
5) Mean value taken for expression in the cases of multiple Gene Symbols.

### Molecular subtyping of oxidative stress-related genes

Clustering and subtyping of the samples were achieved using ConsensusClusterPlus (Wilkerson and Hayes, 2010). To obtain molecular subtypes, expression of cellular senescence-correlated genes were utilized. “KM” algorithm and “1—Pearson correlation” was the metric distance in performing 500 bootstraps. Each bootstrapping contained 80% training set patients. Cluster number was from 2 to 10. Molecular subtypes as well as the optimal classification were obtained through calculation of consistency matrix and consistency cumulative distribution function.

### Risk model

1) Among subtypes, differentially expressed genes (DEGs) were identified by the previously identified molecular subtypes, and we used the Limma package to calculate genes differentially expressed between C1 vs. Other, C2 vs. Other and C3 vs. Other in the TCGA-PAAD cohort (Ritchie et al., 2015).
2) Selection of differentially expressed genes of prognostic significance (p < 0.01).
3) Furthermore, genes were reduced by lasso regression (Tibshirani, 1997) to obtain prognostically significant genes associated with the oxidative stress phenotype.
4) Risk modeling, the formula RiskScore = Σβi × Expi, where Expi is gene expression of the prognostic-related gene with features of the oxidative stress phenotype, and *β* is corresponding gene lasso cox regression coefficient, was used to calculate the risk score for each patient. Then zscore was performed, and patient classification into low- and high- RiskScore groups was conducted under the threshold “0”. To draw raw curves for prognostic analysis, KM method was used and the log-rank test determined difference significance.

### Gene set enrichment analysis (GSEA)

To explore pathways of different biological processes, we used “GSEA” based on all the candidate gene sets in Hallmark database for pathway analysis in different subtypes (Liberzon et al., 2015). Significant enrichment was when false discovery rate (FDR) < 0.05. Ferroptosis pathway were from “WP_FERROPTOSIS” in MSigDB database; autophagy pathway were from “GOBP_REGULATION_OF_AUTOPHAGY” in MSigDB database; from Liu et al. (Liu et al., 2020), we obtained inflammatory signature-related gene set; angiogenesis-related gene set were from Masiero et al. (Masiero et al., 2013).

### Protein-protein interaction (PPI) analysis

PPI networks were produced. The DEGS between subtypes were entered into the STRING online tool (https://string-db.org/), and in Cytoscape (version 3.9.1) software visualization of the PPI networks were done. Next, module analysis of the PPI networks was performed using the Molecular Complex Detection (MCODE) tool of Cytoscape software (Bader and Hogue, 2003).

### Calculation of TME cell invasion

In PAAD, CIBERSORT algorithm (https://cibersort.stanford.edu/) was introduced to quantify relative abundance of 22 immune cells (Newman et al., 2015). ESTIMATE software was applied for the calculation of immune cells proportion, followed by comparison of immune cell infiltration using Wilcoxon test (Runa et al., 2017).

### Correlation analysis of risk score and drug sensitivity

Drug sensitivity data of about 1,000 cancer cell lines were retrieved from Genomics of Drug Sensitivity in Cancer (GDSC) (http://www.cancerrxgene.org) (Yang et al., 2013). Area under ROC curve (AUC) for each antitumor drug served as an indicator for drug response in cancer cell lines. To calculate the association of AMrs scores with drug sensitivity, Spearman correlation analysis was carried out, we considered | Rs |> 0.1. FDR was adjusted by Benjamini and Hochberg, a significant correlation was defined when FDR was less than 0.01.

## Results

### Molecular subtyping based on oxidative stress-associated genes

**FIGURE 1:**
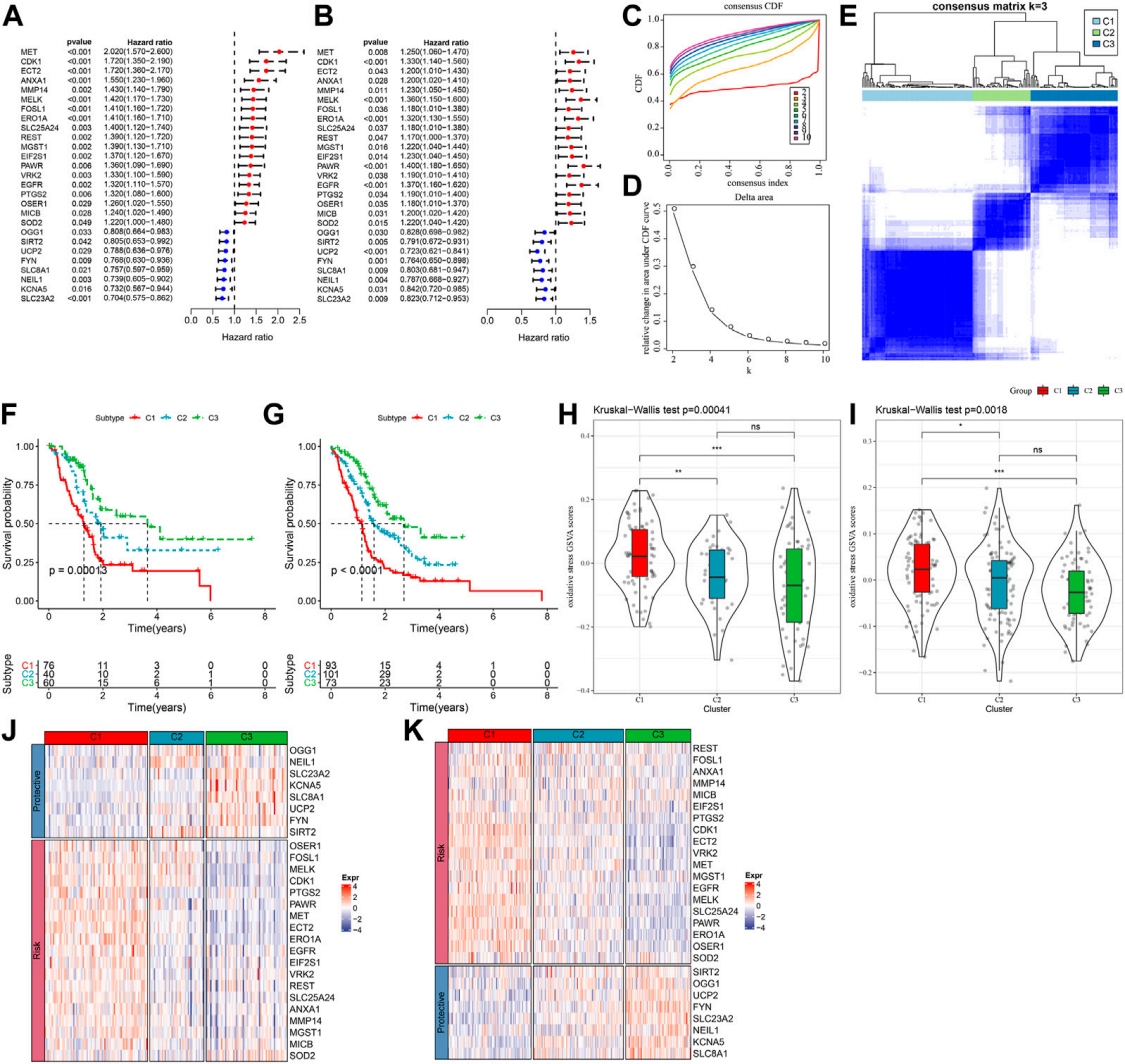
Three pancreatic cancer molecular subtypes based on oxidative stress-related genes. **(A)** In the TCGA-PAAD cohort, the forest plot of 27 prognostically significant oxidative stress genes; **(B)** The forest plot of 27 prognostically significant oxidative stress genes in the PACA-AU cohort; **(C)** CDF curves of TCGA-PAAD samples; **(D)** CDF Delta area curves of the samples, with the horizontal axis indicating the number of categories k and the vertical axis indicating the relative change in area under the CDF curve; **(E)** At consensus k = 3, the heat map of clustered samples; **(F)** KM curve of the prognosis of three subtypes of the TCGA-PAAD samples; **(G)** KM curve of the prognosis of three subtypes of the PACA-AU samples; **(H)** Differences in “oxidative stress ssGSEA scores” among the TCGA-PAAD molecular subtypes; **(I)**Differences in “oxidative stress ssGSEA scores” among the PACA-AU molecular subtypes; **(J)** Heat map of prognostic significant oxidative stress-related genes in TCGA-PAAD subtypes; **(K)** Heat map of prognostic significant oxidative stress-related genes in PACA-AU subtypes.

The expression pattern of oxidative stress-related genes pancreatic cancer samples in the TCGA-PAAD and PACA-AU datasets with clinical information was determined *via* univariate Cox regression. A total of 27 oxidative stress genes showing significant prognosis in both pancreatic cancer datasets were screened. Univariate cox analysis of these 27 genes in TCGA-PAAD and PACA-AU filtered 19“risk genes” and 8“protective genes” (Figures 1A,B). Next, we classified patients by consistent clustering based on 27 prognostically significant oxidative stress gene expression profiles, and according to the cumulative distribution function (CDF), determined the optimal number of clusters. From CDF Delta area curve, we could see that the Cluster selection of 3 had more stable clustering results (Figures 1C,D), and three molecular subtypes (C1, C2, C3) were categorized under k = 3 (Figure 1E). Furthermore, we analyzed their prognostic characteristics and significant differences in prognosis (Figure 1F). Generally, the prognosis of C3 was better in contrast to a worse prognosis of C1. Also, this result was validated in the PACA-AU cohort (Figure 1G).

Analysis on the “oxidative stress ssGSEA scores” for each pancreatic cancer patient in the TCGA-PAAD cohort showed that the C1 subtype had higher “oxidative stress ssGSEA scores” and it was the lowest in C3 (Figure 1H), noticeably, C1 presented activated oxidative stress. We also compared the expression differences of 27 oxidative stress genes in different molecular subtypes (Figure 1J). C1 subtype showed an overall high-expressed “Risk” genes, while in the C3 subtype, the “Protective” gene was high-expressed. This phenomenon was also observed in the PACA-AU cohort (Figures 1I,K).

### Genomic landscape between molecular subtypes

**FIGURE 2:**
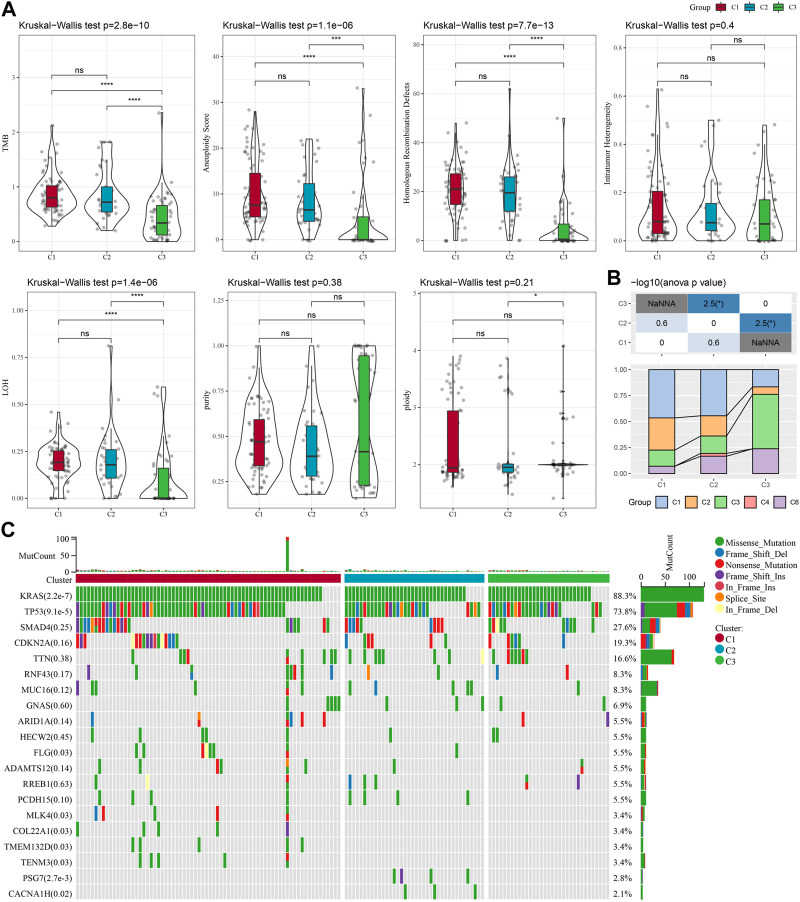
In TCGA-PAAD cohort genomic alterations of molecular subtypes. **(A)** Comparison of Aneuploidy Score, LOH, tumor 25 burden, Intratumor Heterogeneity, ploidy, Homologous Recombination Defects, purity in TCGA-PAAD subtypes; **(B)** Comparing our molecular subtypes to the other six existing immune molecular subtypes; **(C)** Chi-square test on the somatic mutations in the three molecular subtypes. *p < 0.05; **p < 0.01; ***p < 0.001; and ****p < 0.0001.

To further investigate the potential molecular mechanisms underlying the classification of oxidative stress subtypes, we explored genomic alteration differences among these three TCGA cohort molecular subtypes. Here, information of molecular signature of TCGA-PAAD was acquired from a previous pan-cancer study (Thorsson et al., 2018). The Anenploidy Score, loss of heterogeneity (LOH), tumor mutation burden (TMB), Homologous Recombination Defects all differed greatly among the three subtypes. It has been observed that the C1 subtype had higher levels of these four indicators (Figure 2A). In addition, in this study, according to 160 different immune signatures, five molecular subtypes of PADD were categorized, among which the most favorable prognosis was the immunoassay subtype C3. Then, comparison of the current molecular subtypes were compared with the five immune molecular subtypes showed that our C3 subtype was more occupied by the immune molecular subtype C3, which coincided with the most favorable prognosis of our molecular subtype C3 (Figure 2B). The top significant 20 genes were shown (Figure 2C). It could be seen that genes such as KRAS and TP53 had significantly different mutation frequencies between the three molecular subtypes.

### Immune characteristics between molecular subtypes and differences in immunotherapy/chemotherapy

**FIGURE 3:**
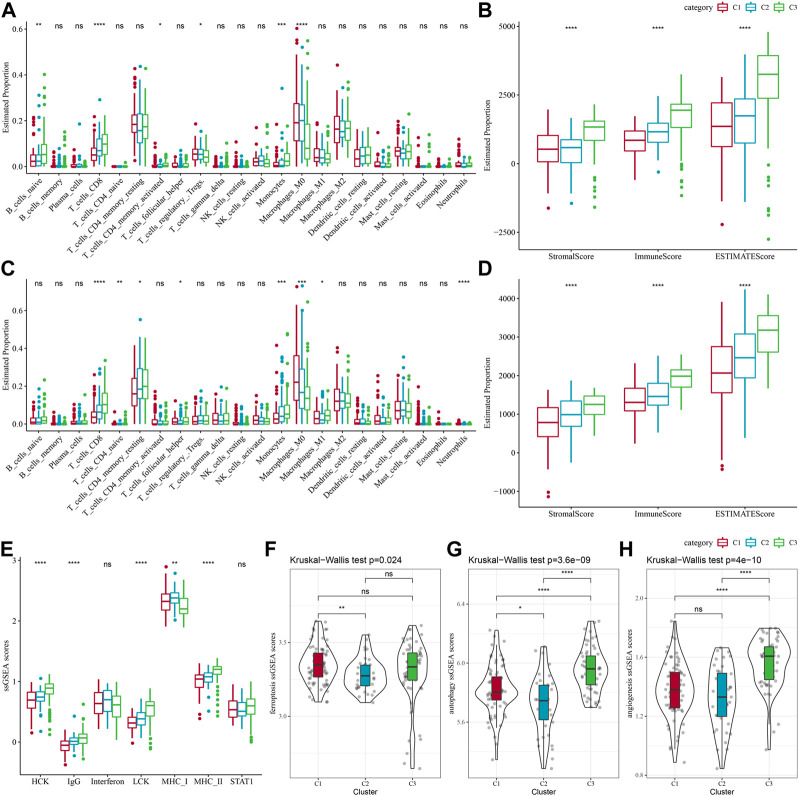
Immune characteristics of different subtypes. **(A)** TCGA-PAAD molecular subtypes varied in the differences of 22 immune cell scores; **(B)** TCGA-PAAD molecular subtypes varied in the differences of ESTIMATE immune infiltration; **(C)** PACA-AU molecular subtypes varied in the differences of 22 immune cell scores; **(D)** PACA-AU molecular subtypes varied in the differences of ESTIMATE in immune infiltration; **(E)** TCGA-PAAD molecular subtypes varied in the differences of scores of seven inflammation-related gene clusters; **(F)** TCGA-PAAD molecular subtypes varied in the differences of ferroptosis pathway; **(G)** TCGA-PAAD molecular subtypes varied in the differences in scores of autophagy pathway; **(H)** TCGA-PAAD molecular subtypes varied in the differences in scores of angiogenesis-related genes; *p < 0.05; **p < 0.01; ***p < 0.001; and ****p < 0.0001.

Between different molecular subtypes, differences in the PAAD immune microenvironment was further explored by assessing immune cell infiltration in patients in TCGA-PAAD and PACA-AU cohorts based on gene expression in the immune cells. Relative abundance of 22 immune cell types was determined using CIBERSORT, and in the TCGA-PAAD cohort we found that six immune cell types (Mcrophages, CD8 T cells, naive B cells, Monocytes, memory CD4 T cells, regulatory T cells, Macrophages M0) differed significantly between subtypes, and T_cells_CD8 and Monocytes were enriched in the C3 subtype (Figure 3A). Immune cell infiltration was assessed using ESTIMATE. In the TCGA cohort, the three subtypes differed significantly in distribution in the StromalScore, ImmuneScore and ESTIMATEScore, and the highest score was in the C3 subtype but the lowest was in the C1 subtype (Figure 3B). We also analyzed the PACA-AU data set and found that eight immune cell types, including resting memory CD4 T cells, M0 Macrophages, naïve CD4 T cells, M1 Macrophages, CD8 T cells, helper follicular T cells, Monocytes, Neutrophils, in the PACA-AU cohort differed significantly between subtypes (Figure 3C). Furthermore, immune cell infiltration in the PACA-AU cohort was consistent with the TCGA-PAAD cohort (Figure 3D).

Some studies have reported that oxidative stress and inflammation are intertwined processes in disease progression and response to therapy by interfering with multiple signaling pathways. Here, the enrichment scores of seven metagenes clusters were greatly different in the three molecular subtypes, with the exception of Interferon, STAT1, and the remaining five metagenes clusters, and overall, the C3 subtype had higher inflammatory activity (Figure 3E). In addition, it has been reported that ferroptosis from oxidative stress and inflammation plays a key role in the pathogenesis of cardiovascular diseases (Yu et al., 2021), such as stroke, vascular sclerosis, heart failure, ischemia-reperfusion injury. Thus, comparison on the differences in ferroptosis scores between the three subtypes has demonstrated significant distributional differences between C1 and C2 subtypes, with C2 subtype having a lower ferroptosis score (Figure 3F). In addition, a study reported that inflammation stimulates excessive autophagy or severe oxidative stress could result in autophagy-dependent cell death (Cai et al., 2018). The autophagy scores for the subtypes (Figure 3G) were significantly different between the three subtypes, with the C3 subtype having a higher autophagy score. Additionally, we found statistically significant differences in angiogenesis between C2 and C3 subtypes and between C1 and C3 subtypes, with C3 subtype having the highest score (Figure 3H).

### Immunotherapy and drug sensitivity differences between molecular subtypes

**FIGURE 4:**
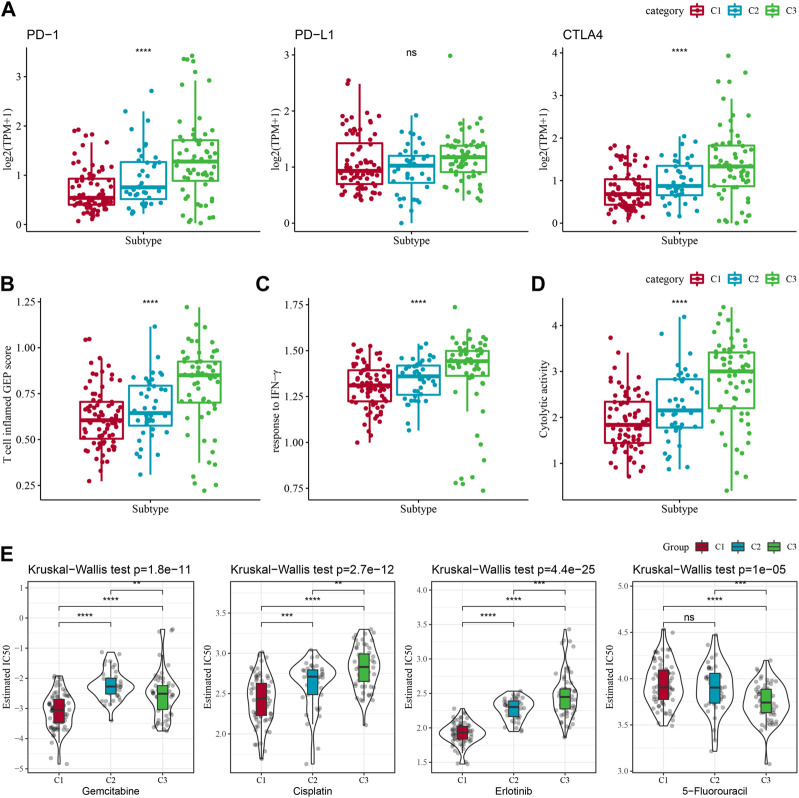
Differences in treatment sensitivity among the molecular subtypes. Among different molecular subtypes, **(A)** Differences in “T cell inflamed GEP score”; **(B)** differences in “response to IFN-γ”; **(C)** Differences in “response to IFN-γ". **(B)** Differences in “response to IFN-γ”; **(C)** Differences in immune checkpoint gene expression; **(D)** Differences in “Cytolytic activity”; **(E)** Box plots of IC50 of cisplatin, gemcitabine, 5-fluorouracil, erlotinib in TCGA-PAAD; *p < 0.05; **p < 0.01; ***p < 0.001; and ****p < 0.0001.

Some sample compounds were examined because immune checkpoint blockade (ICB) cancer treatment works by suppressing important immune checkpoints. Among the three subtypes, CTLA4 and PD-1 were differentially distributed, and C3 was significantly more high-expressed, while PD-L1 was not differentially expressed (Figure 4A). We also applied the “T-cell-inflamed GEP score” to assess the predictive potential of different molecular subtypes in immunotherapy for cancers. It could be observed from Figure 4B, the C3 subtype had a noticeably higher “T-cell-inflamed GEP score”. Considering that IFN-γ is a cytokine with a key role in anti-cancer immunity and immunomodulation (Rydyznski Moderbacher et al., 2022), our analysis revealed that in the C3 subtype the IFN-γ response was significantly enhanced (Figure 4C). Additionally, we also found that Cytolytic activity (CYT) scores, which reflects cytotoxic effects, were significantly higher in C3 subtypes compared with other subtypes (Figure 4D).

Additionally, response of the molecular subtypes in the TCGA-PAAD cohort to the conventional chemotherapeutic agents such as Gemcitabine, Erlotinib, Cisplatin, 5-Fluorouracil were analyzed, and found that C1 was more sensitive to Gemcitabine, Erlotinib, Cisplatin (Figure 4E), while C3 was more sensitive to 5-Fluorouracil.

### Differential functional analysis between the molecular subtypes

Limma package was used to determine DEGs. There is 64 DEGs in C1vs. other in TCGA-PAAD dataset and PACA-AU dataset, 137 DEGs in C3 vs. other in TCGA-PAAD dataset and PACA-AU dataset. After union analysis, 144 DEGs were obtained. Functional enrichment analysis was conducted on the DEGs among the subtypes. The enrichment results of GO and KEGG pathways on the DEGs in the “C1” subtype demonstrated that the DEGs had significant enrichment in some biological functions than cellular communication (Supplementary Figure S1A). However, in “C3” subtype these DEGs were significantly enriched to some immune-related biological pathways (Supplementary Figure S1B). To better investigate the interactions among these DEGs, the STRING online tool for developing a PPI network (Supplementary Figure S1C) was applied. In addition, two important modules in the PPI network were determined based on the module analysis (Supplementary Figure S1D).

### Identification of key genes for the oxidative stress phenotype

**FIGURE 5:**
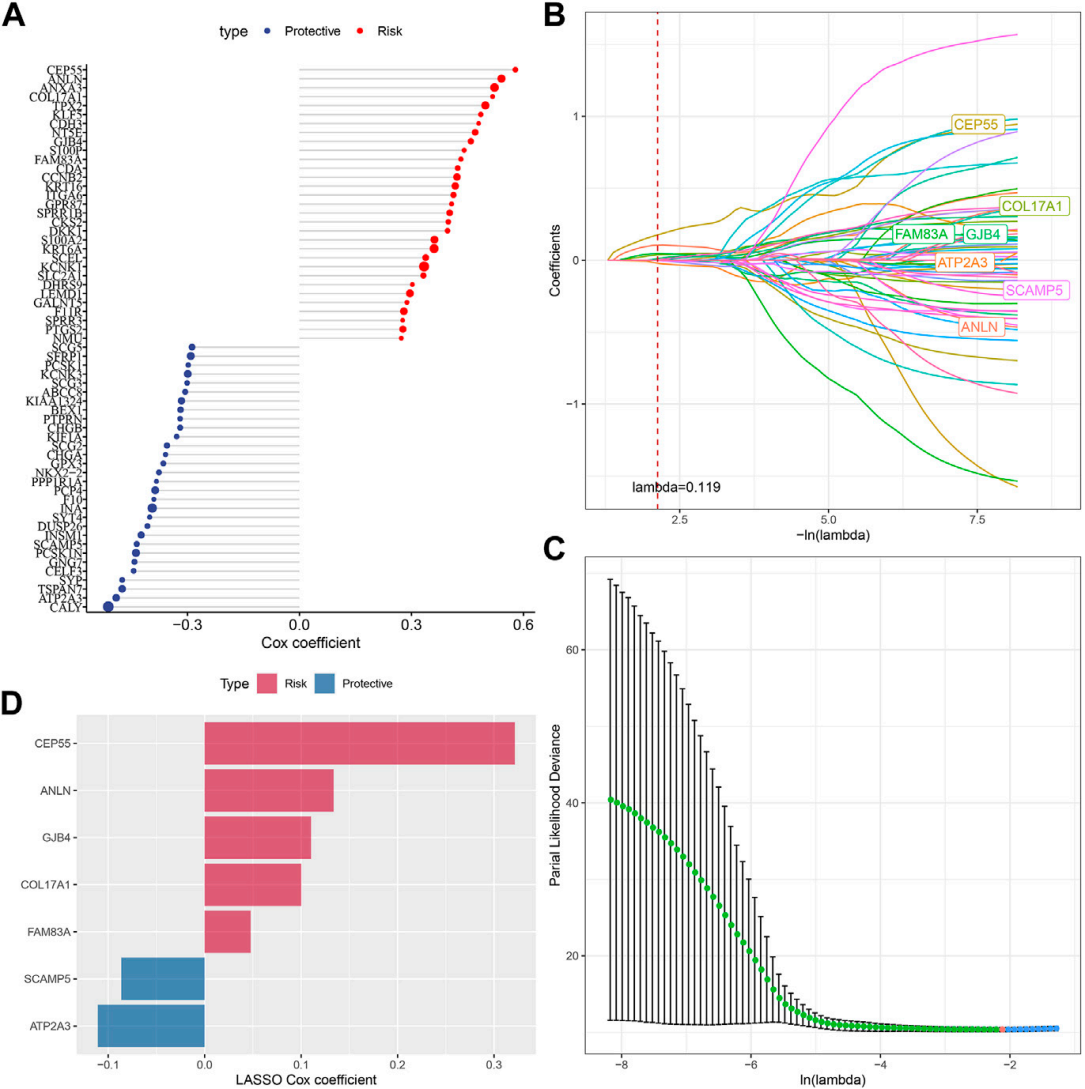
Screening of genes correlated with oxidative stress that affect prognosis. **(A)** Totally 61 candidates were screened from all the DEGs; **(B)** With the change of lambda, trajectory of each independent variable was shown; **(C)** Confidence interval under lambda; **(D)** Oxidative stress-related prognostic gene markers and the distribution of LASSO coefficients.

Next, we performed univariate COX regression analysis on 144 DEGs among the subtypes and identified 61 genes showing great prognostic significance (*p* < 0.01), including 31“Risk” and 30“Protective” genes (Figure 5A). PPI network analysis indicated that these genes are related to each other (Supplementary Figure S2). To further compress these 61 genes in the risk model, Lasso regression was used. Independent variable’s trajectory is shown in Figure 5B. The number of independent variable coefficients close to zero likewise showed a progressive increase as the lambda gradually increased. Moreover, 10-fold cross-validation was applied to develop a model and to analyze confidence intervals under each lambda (Figure 5C). When lambda = 0.119, the model was optimized, therefore, 7 genes at lambda = 0.119 were determined in this study as the genes related to oxidative stress to affect patients prognosis (Figure 5D). These genes included ATP2A3, ANLN, GJB4, FAM83A, CEP55, COL17A1, and SCAMP5. The formula as followed: RiskScore = + 0.134*ANLN-0.086*SCAMP5+0.048*FAM83A-0.111*ATP2A3+0.322*CEP55 + 0.11*GJB4+0.1*COL17A1 Single-cell division TISCH2 (http://tisch.comp-genomics.org/home/) analyzed the expression distribution of seven genes in multiple single-cell data of pancreatic cancer, and the results showed that the expression of COL17A1 and FAM83A genes in malignant cells was significantly higher than that in other cell types (Supplementary Figure S3).

### The performance and validation of prognostic model

**FIGURE 6:**
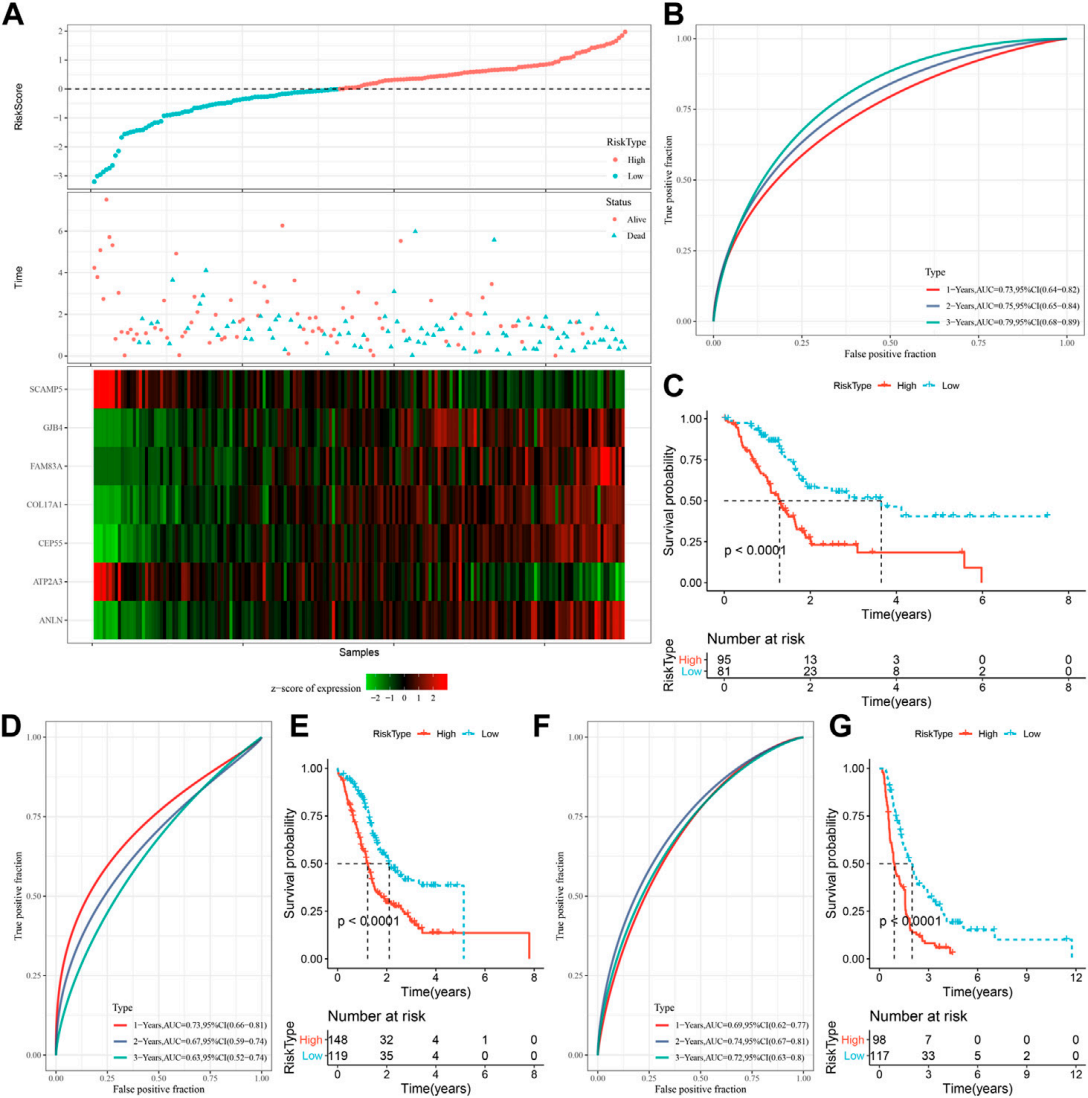
Generation and evaluation of risk score models using 7 genes related to oxidative stress. **(A)** RiskScore, expression of oxidative stress-related prognostic genes and survival time and status in TCGA dataset; **(B)** RiskScore classification in TCGA dataset and ROC and AUC curves; **(C)** Distribution of KM survival curve of RiskScore in TCGA dataset; **(D, E)** ROC curve and KM survival curve distribution of RiskScore in PACA-AU cohort; **(F, G)** ROC curves and KM survival curves of RiskScore in the PACA-CA cohort.

The expression and coefficients of seven prognostic genes were used to construct a clinical prognostic model and for calculating and ranking the risk values of TCGA-PAAD samples. According to the cut-off, we divided 81 samples into “Low-risk” group and 95 samples were in the “High-risk” group. The prognosis prediction at 1, 2, and 3 years (s) was further analyzed for its classification efficiency (Figure 6B), respectively. The model demonstrated a high area under the AUC line (1-Year, AUC = 0.73; 2-Year, AUC = 0.75; 3-Year, AUC = 0.79). KM curves were plotted and a highly significant difference was shown between the two RiskScore groups (*p* < 0.0001), with the “Low-risk” group showing a significantly better prognostic outcome than “High-risk” group (Figure 6C). To confirm the robustness of the clinical prognostic model, we performed validation in 2 additional independent pancreatic cancer cohorts (PACA-AU, PACA-CA), and it can be seen that in the validation cohort showed similar results to the training set, with the “Low-risk” group showing a significantly better prognostic outcome than “High-risk” group (Figures 6D–G).

### The RiskScore on the subtypes and various clinicopathological features

To assess the correlation of PAAD clinical features with RiskScore, the differences in RiskScore between different TNM grades and clinical stages in the TCGA-PAAD and PACA-AU datasets were studied. Samples with higher clinical grades showed higher RiskScore. Also, C1 subtypes had the highest RiskScore but C3 subtypes had the lowest RiskScore (Supplementary Figures S4A,C). Additionally, difference comparison between RiskScore groups and molecular subtypes was conducted, showing a majority of “C1” or “C2” patients in the “high-risk” group (Supplementary Figures S4B,D). Moreover, the prognosis of TCGA-PAAD between the low- and high-risk groups in relation to clinicopathological characteristics was explored, and our risk grouping was equally effective across clinical subgroups, with the “Low-risk” group showing a significantly better prognosis, demonstrating the reliability of our risk grouping (Supplementary Figures S4E). In addition, the correlation analysis of spearman between expression levels and methylation on 6 genes except ANLN gene showed a negative phenomenon but had a positive with SCAMP5 gene (Supplementary Figures S5).

### Immune infiltration/pathway characteristics between RiskScore subgroups

**FIGURE 7:**
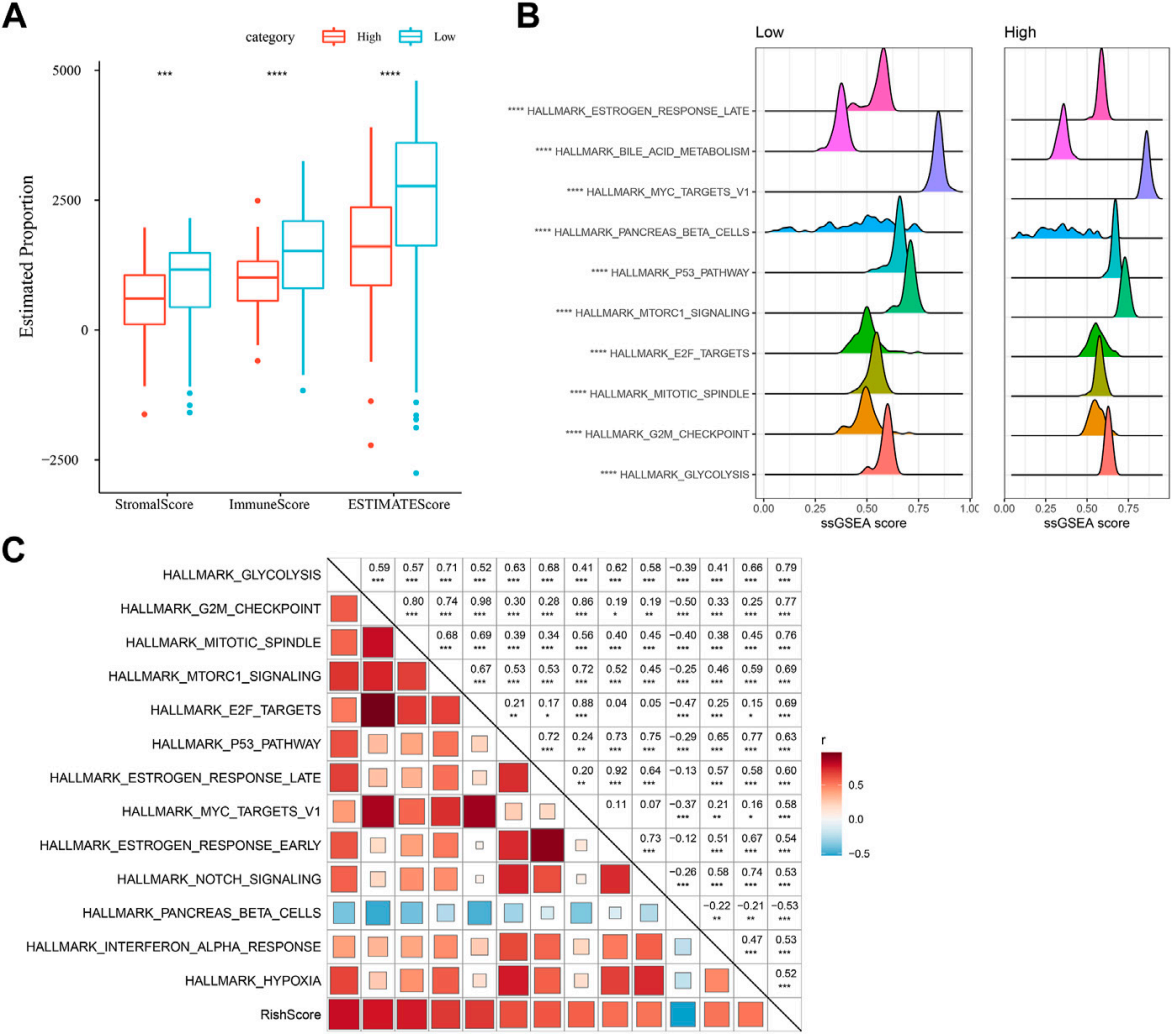
Immunology and pathway between different RiskScore subgroups. **(A)** ESTIMATE software was applied to determine immune cell components in the TCGA database; **(B)** The top 10 pathways showing the greatest significant differences between Low-risk and high-risk groups; **(C)** Correlation analysis results on the RiskScore and KEGG pathways scored greater than 0.5; *p < 0.05; **p < 0.01; * **p < 0.001; and ****p < 0.0001.

Differences in immune microenvironment in the RiskScore subgroups were studied, we used ESTIMATE to assess immune cell infiltration (Figure 7A), and observed that the “Low-risk” group was significantly higher in immune cell infiltration. The most significant top 10 pathways showing differences between the high- and low-risk groups are shown in Figure 7B. It can be seen that high-RiskScore was significantly enriched to some cell cycle-related pathways such as G2M_CHECKPOINT, MTOTIC SIGNSLING, and E2F TARGETS. Furthermore, association of the RiskScore with the enrichment scores of these functions was analyzed, with the functions showing a correlation greater than 0.5 being identified. Figure 7C manifests a positive correlation of the RiskScore with cell cycle-related pathways.

### Differences in chemotherapy/immunotherapy among the RiskScore subgroups

**FIGURE 8:**
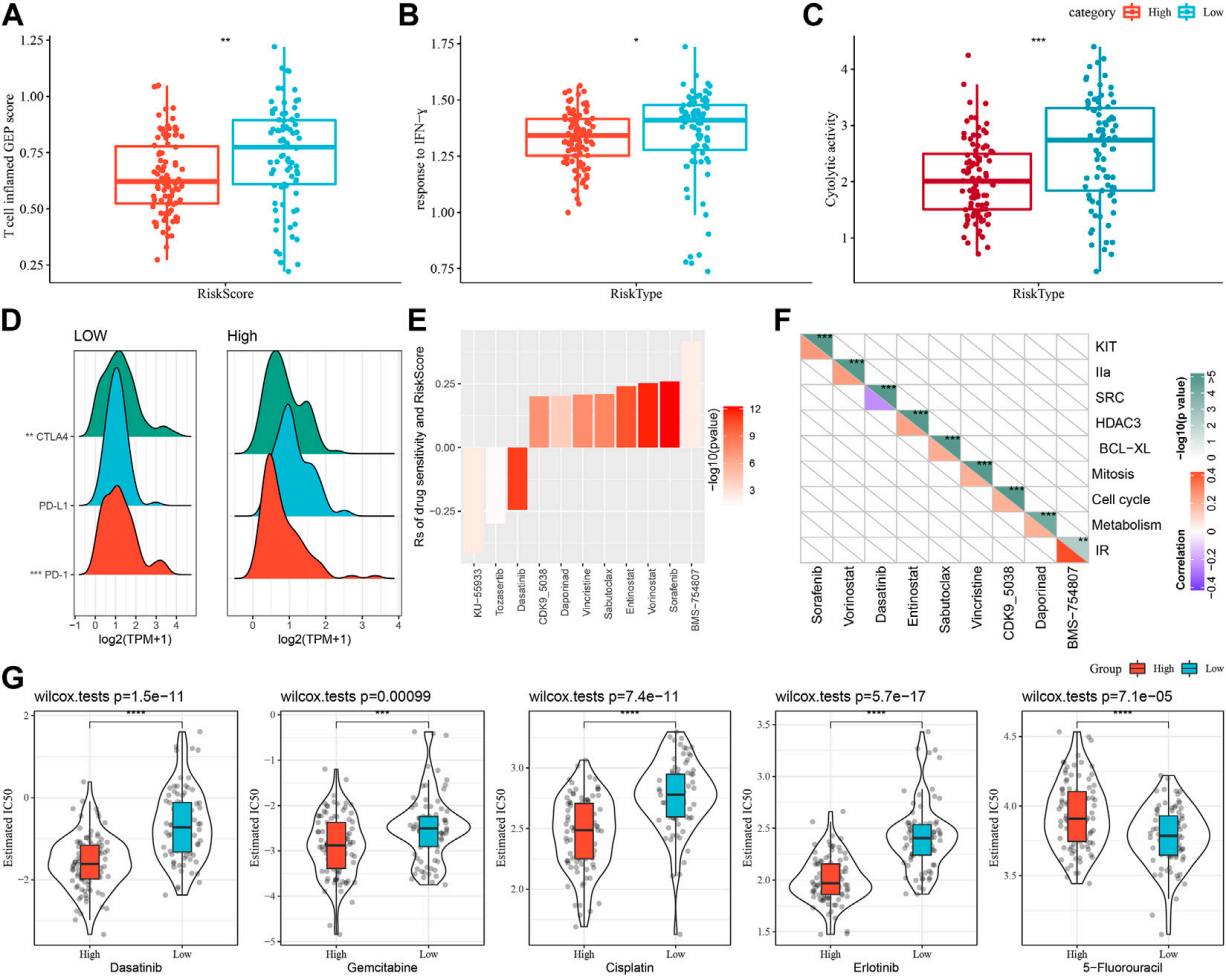
The prognostic risk models in predicting patients’ benefit from immunization/chemotherapy. **(A)** Differences in “T cell inflamed GEP score” between subgroups; **(B)** Differences in “response to IFN-γ” between subgroups; **(C)** Differences in “C-γ” between subgroups. **(C)** Difference of “Cytolytic activity” between different subgroups; **(D)** Difference of immune checkpoint gene expression between different subgroups; **(E)** Spearman analysis was conducted for correlation analysis on drug sensitivity and RiskScore, with each column representing a type of drug. Correlation significance is reflected in color brightness. The correlation of RiskScore with drug sensitivity (Rs < 0) or drug resistance (Rs > 0) was reflected in the height of a column. **(F)** The horizontal axis is the drug name and the vertical axis is the signaling pathway targeted by the drug. The signaling pathway targeted by the drug is sensitive to RiskScore (blue); **(G)** Box plots of IC50 estimates for dasatinib, gemcitabine, cisplatin, erlotinib and 5-fluorouracil in TCGA-PAAD; *p < 0.05; **p < 0.01; ***p < 0.001; and ****p < 0.0001.

First, we used the “T-cell-inflamed GEP score” to assess the prediction potential of the different RiskScore subgroups in cancer immunotherapy (Figure 8A), and the results showed that in the low-RiskScore group the “T-cell-inflamed GEP score” was significantly higher. Further analysis on the response to IFN-γ in both groups revealed that the response to IFN-γ was significantly enhanced in the low-RiskScore group (Figure 8B). Moreover, the CYT score had cytotoxic effect, and it was significantly lower in the high-RiskScore group (Figure 8C). Some representative immune checkpoint molecules were significantly high-expressed CTLA4, PD-1 in the low-RiskScore group, while PD-L1 was not differentially expressed between molecular subtypes (Figure 8D).

The effect of RiskScore on drug response was analyzed based on the relationship between RiskScore and cancer cell lines’ response to drugs. There were 11 drug sensitivities in the GDSC database showing significant correlation with RiskScore. There were three drug sensitivities showing a negative correlation with RiskScore, namely, KU-55933, Tozasertib, Dasatinib (Figure 8E). Furthermore, we also analyzed the signaling pathways of the genes targeted by these drugs, which mainly target the SRC pathway (Figure 8F).

Moreover, response degree of the TCGA-PAAD subtypes to chemotherapeutic agents (Gemcitabine, Erlotinib, Cisplatin, 5-Fluorouracil, and Dasatinib) was studied. We found that the High-risk group responded to Gemcitabine, Cisplatin, Erlotinib and Dasatinib. Overall, High-risk group showed a higher sensitivity to Gemcitabine, Cisplatin, Erlotinib and Dasatinib. Low-risk group had higher sensitivity to 5-Fluorouracil (Figure 8G).

### RiskScore in combination with clinicopathological features to improve survival prediction and the prognostic models

**FIGURE 9:**
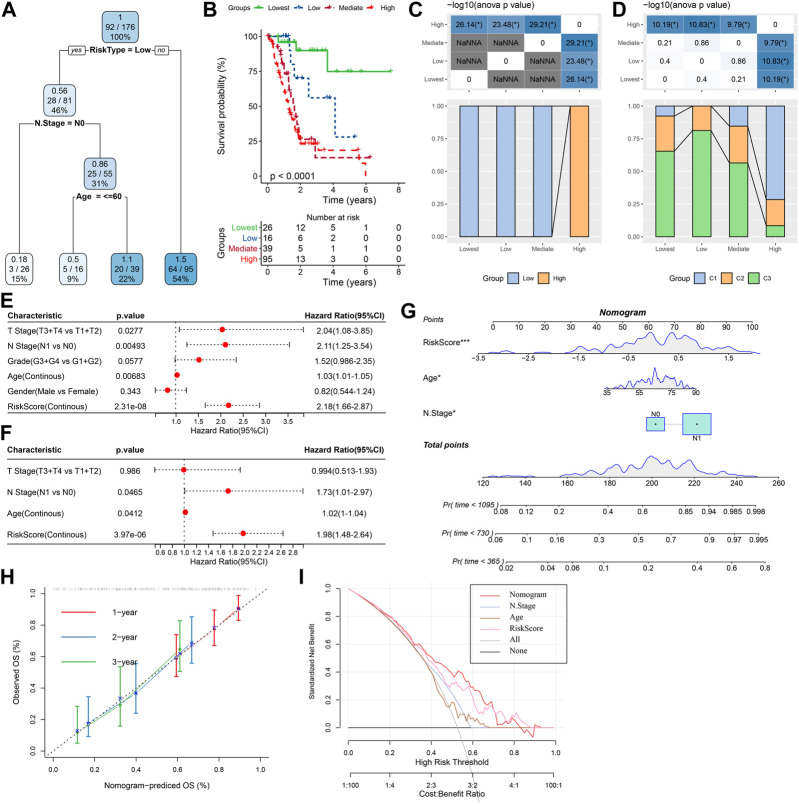
The nomogram of prognostic risk models with clinicopathological features. **(A)** To optimize risk stratification, patients with full-scale annotations including TNM Stage, age, gender, and RiskScore were enrolled for developing a survival decision tree; **(B)** Risk subgroups showed significant overall survival differences; **(C, D)** Comparative analysis between different subgroups; **(E, F)** RiskScore and clinicopathological **(E, F)** Univariate and multifactorial Cox analysis on clinicopathological features and RiskScore; **(G)** The nomogram model; **(H)** Calibration curves for 1, 3, and 5 years (s) of the nomogram; (H:Decision curve for the columnar graph; **(I)** The most powerful capacity of the nomogram for survival prediction when compared with other clinicopathological features. *p < 0.05; **p < 0.01; ***p < 0.001; and ****p < 0.0001.

For the TCGA-PAAD cohort (Figure 9A), only RiskType, and Age, N Stage remained in the decision tree that was originally established with TNM Stage, gender, pathology information and RiskScore, patient age, and it identified four different risk subgroups (Lowest, Low, Mediate, High). Among them RiskType was the parameter of the greatest impact. The four risk subgroups showed significant difference in overall survival, with the “Lowest” group having the optimal prognosis and the “High” group having the worst prognosis (Figure 9B). Patients in the risk subgroups were all “Low","Lowest”, and “Mediate” Low-risk group patients (Figure 9C). In addition, “High-risk” group showed more distribution of our defined molecular subtypes C1 and C2 (Figure 9D). From Figures 9E,F, the most significant prognostic factor was the RiskScore. To quantify survival probability of PAAD patients and the risk assessment, other clinicopathological characteristics were combined with RiskScore for nomogram development (Figure 9G), here, the RiskScore demonstrated the greatest impact on predicting patients’ survival. Furthermore, we evaluated the model prediction accuracy with calibration curve (Figure 9H). At the calibration points of 1, 2, 3 years (s), the prediction calibration curves almost completely encircled the standard curve, indicating good prediction accuracy. We also assessed model reliability by decision curve analysis, and in comparison to the extreme curves, benefit of both RiskScore and Nomogram was significantly greater and the two showed a stronger survival prediction (Figure 9I).

Herein, we selected three risk models from previous studies (5-gene signature (Yan) (Yan et al., 2022), 3-gene signature (Yang) (Yang et al., 2022) and 9-gene signature (Liu) (Liu et al., 2022)) to compare with our model. In order to make the model have a certain comparability, the same method is used to calculate the sample risk score according to the corresponding genes in the three models, and zscore is performed for Riskscore. After zscore, the samples with Riskscore greater than zero are divided into high-risk group and those with riskscore less than zero are divided into low-risk group.

ROC analysis showed that the AUC value was lower than that in our model (Supplementary Figures S6A–C). The C-index in our model was higher than that in other 3 models (Supplementary Figure S6D).

## Discussion

Pancreatic cancer shows a significantly poor prognosis with a 5-year survival chance of approximately 5% (Ilic and Ilic, 2016). Accurate prognostic evaluation enables patients suffering from PAAD to benefit more from effective treatments such as more intensive surgery, targeted molecular therapies, neoadjuvant therapy, immunotherapy, radiotherapy, chemotherapy. Thus, treatment could be personalized to individual patient for improving prognosis. In the early diagnosis of highly heterogeneous PAAD, molecular prognostic markers are potentially valuable, which at the same time could help overcome the impediment of heterogeneity. Multiple molecular markers increase the accuracy than single molecular markers in reflecting pancreatic cancer prognosis, the progression of which is in a complex network involving different signaling pathways. Wu et al. identified a 9-gene signature and also developed a prognostic nomogram that could reliably predict PAAD overall survival (Wu et al., 2019). Weng et al. established a multi-omics perspective consisting of 3 mRNAs, 3 miRNAs, 60 lncRNAs related to PAAD prognosis, and constructed a classifier based on 14 mRNAs with a good predictive function in the cohort and helped to predict PAAD prognosis (Weng et al., 2020). Based on 14 necroptosis-associatedgenes, Wu et al. developed a prognostic model for the diagnosis, prognosis of PAAD and its treatment (Wu et al., 2022). Our work identified the molecular subtypes of PAAD based on oxidative stress due to the non-negligible regulatory impact from oxidative stress plays.

First, we employed oxidative stress-related genes to consistently cluster three stable molecular subtypes, each of which has its own unique prognostic, route, clinical, and immunological properties. Our analysis demonstrated a better prognosis of C3 and a worse one of C1. ROS could cause several types of DNA damage (Ebrahimi et al., 2020). Persistent DNA damage is resulted from the continuous production of ROS and an inflammatory cascade that triggers genomic changes and a tendency to increase epigenetic alterations. The development of cancer may be facilitated by the accumulation of epigenetic changes that disrupt genome-wide cell signaling system and promote malignant transformation (Kgatle et al., 2017). Our comparative analysis of genomic alterations in the three subtypes revealed that the C1 subtype showed higher “TMB”, “Homologous Recombination Defects”, “Aneuploidy Score”, “Intratumor Heterogeneity”, “LOH”. TMB is a sensitive biomarker for screening sensitive responders to immunotherapy and has been shown to be correlated with more significantly with response, with higher blockade of PD-L1 and PD-1 than PD-1 or PD-L1 expression. Mechanistically, high TMB provides more opportunities for “non-me” neoantigen production and activation of immune cell enrichment. Nevertheless, these theories have only been confirmed in some places for immunotherapy of certain tumors, but they may not be applicable to tumors such as pancreatic cancer (Strickler et al., 2021). For immune microenvironment differences, significantly higher immune cell infiltration and “ImmuneScore” were observed in C2 and C3. In addition, as oxidative stress is closely associated with multiple physiological activities, and our analysis demonstrated that C3 subtypes had higher inflammatory activity and autophagy scores and the lowest angiogenic scores. Though many achievements have been made in the immunotherapy of cancer, not all patients can benefit from immunotherapy. Our analysis showed significantly enhanced IFN-γ response and higher “T-cell-inflamed GEP score” in the C3. Additionally, the CYT score, which reflects cytotoxic effects, was noticeably higher in C3 than in other subtypes. In the multimodal treatment of pancreatic cancer, chemotherapy is an important component. Adjuvant chemotherapy can significantly improve disease-free survival and overall survival after curative resection (Springfeld et al., 2019). Analysis on conventional chemotherapeutic drug response showed C1 was more sensitive to Erlotinib, Cisplatin, and Gemcitabine.

Seven key genes (GJB4, CEP55, SCAMP5, ANLN, FAM83A, ATP2A3, COL17A1) associated with oxidative stress phenotypes, were identified. CEP55 plays an important role in cytoplasmic division, tumor stage, aggressiveness, metastasis and poor prognosis in many tumor types such as breast, lung, colon and liver cancers (Jeffery et al., 2016). In many malignancies ANLN is an upregulated actin-binding protein. Wang et al. found that in pancreatic cancer tissues and cell lines, ANLN expression is upregulated and is predictive of a poor PAAD prognosis. ANLN-mediated pancreatic cancer invasion and migration, colony formation, cell proliferation may involve EZH2/miR-218-5p/LASP1 signaling axis (Wang et al., 2019). The gap junction *β*-4 protein is an integral membrane protein member involved in tumorigenesis and may play a role as a tumor promoter (Liu et al., 2019). Moreover, in lung cancer, it has also been found to induce chemoresistance and metastasis *via* Src activation (Lin et al., 2019). As an important component of type I hemibridges (HD), COL17A1 encodes collagen XVII (COL17) (Yodsurang et al., 2017), and has been identified as a marker for pancreatic cancer by Shen et al. (Shen et al., 2017). In a variety of human tumors, family with sequence similarity 83 member A was initially identified by bioinformatics methods as a potential tumor-specific gene with overexpression, including in bladder, lung, testicular, breast cancers, etc. Chen et al. found that in pancreatic cancer FAM83A shows significant overexpression, which promotes CSC-like features by activating Wnt/β-catenin and TGF-β pathways. Therefore they concluded that FAM83A has the potential of acting as a therapeutic target for patients with pancreatic cancer (Chen et al., 2017). SCAMP functions as a post-Golgi transporter protein in all mammalian cells and is an effective prognostic and diagnostic biomarker for pancreatic cancer (Mao et al., 2021). ATP2A3 is a significantly upregulated gene that encodes a Ca2+ -ATPase localized to the ER membrane and is involved in Ca2+ transport (Zhang et al., 2019). Eduardod et al. showed that resveratrol upregulates the expression of the ATP2A3 gene in breast cancer cell lines through an epigenetic mechanism (Izquierdo-Torres et al., 2019).

In spite of this, there are some limitations in this study, which should be verified by PCR and immunohistochemical experiments. We did not consider other factors because the samples lacked necessary clinical follow-up information, especially diagnostic details.

## Conclusion

This paper first identified a novel prognostic risk model consisting of 7 oxidative stress-related genes that well predict PAAD prognosis of PAAD. The 7 genes demonstrated complex molecular functions that remained to be explored further. In addition, this work highlighted the correlation between of the prognosis of PAAD with oxidative stress-related genes. The current findings facilitate personalized treatment for PAAD patients.

## Data Availability

The original contributions presented in the study are included in the article/Supplementary Material, further inquiries can be directed to the corresponding author.
