# Supplementary figures and images for: Establishment of a 7-gene prognostic signature based on oxidative stress genes for predicting chemotherapy resistance in pancreatic cancer
Source: Front Pharmacol. 2023 Apr 17;14:1091378. doi: 10.3389/fphar.2023.1091378 (PMC10149707; doi:10.3389/fphar.2023.1091378)

ANLN

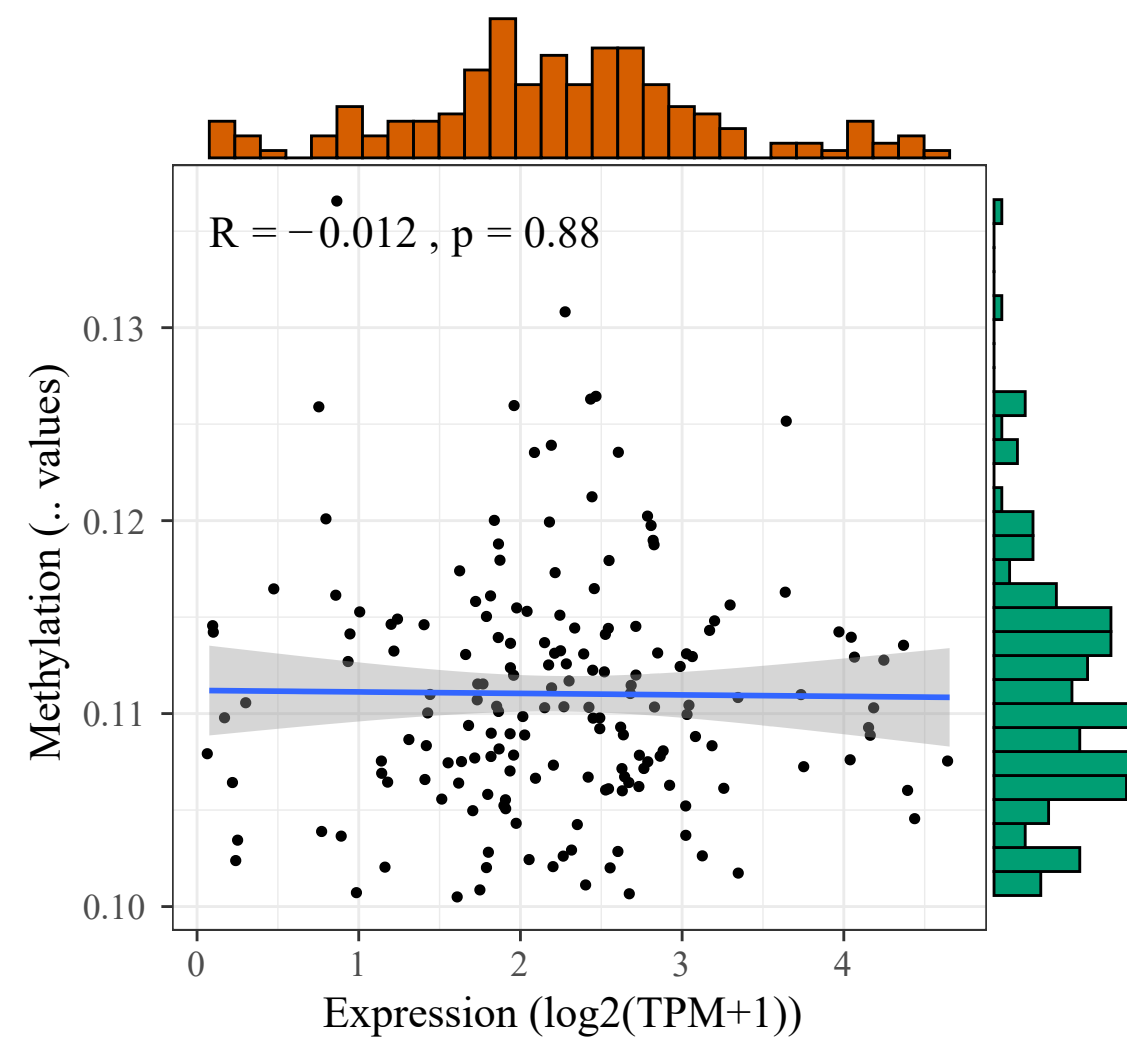

SCAMP5

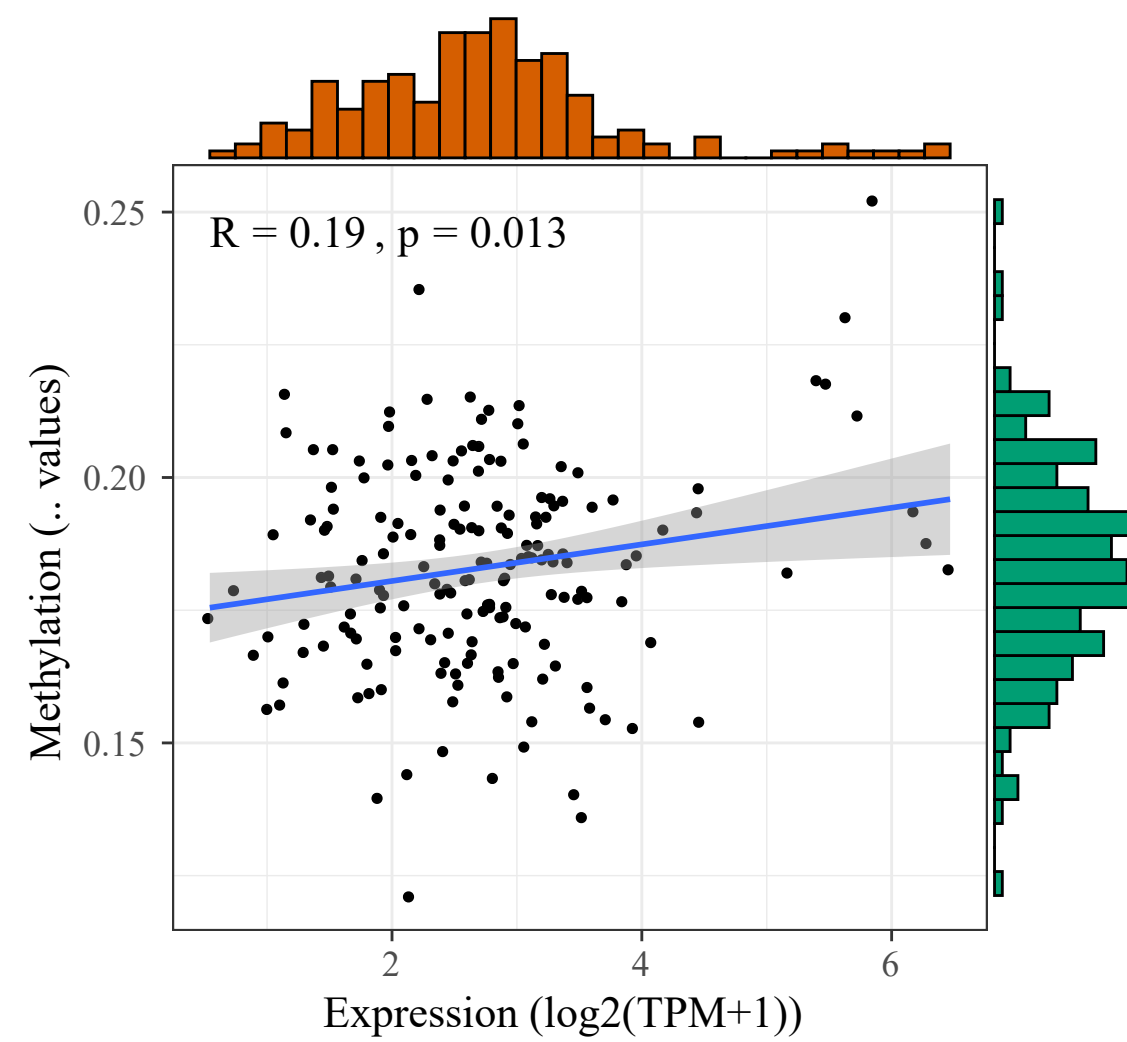

FAM83A

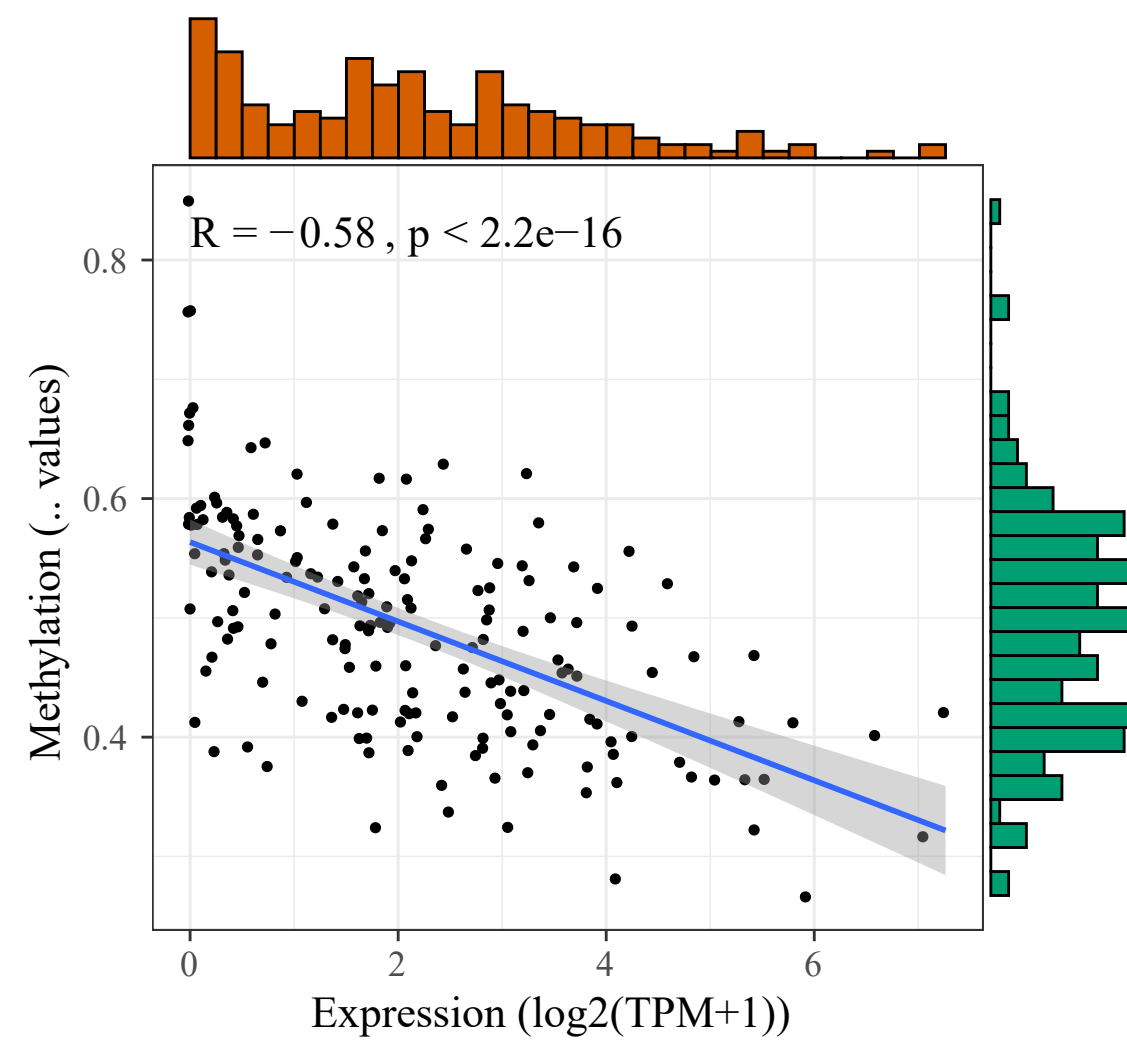

ATP2A3

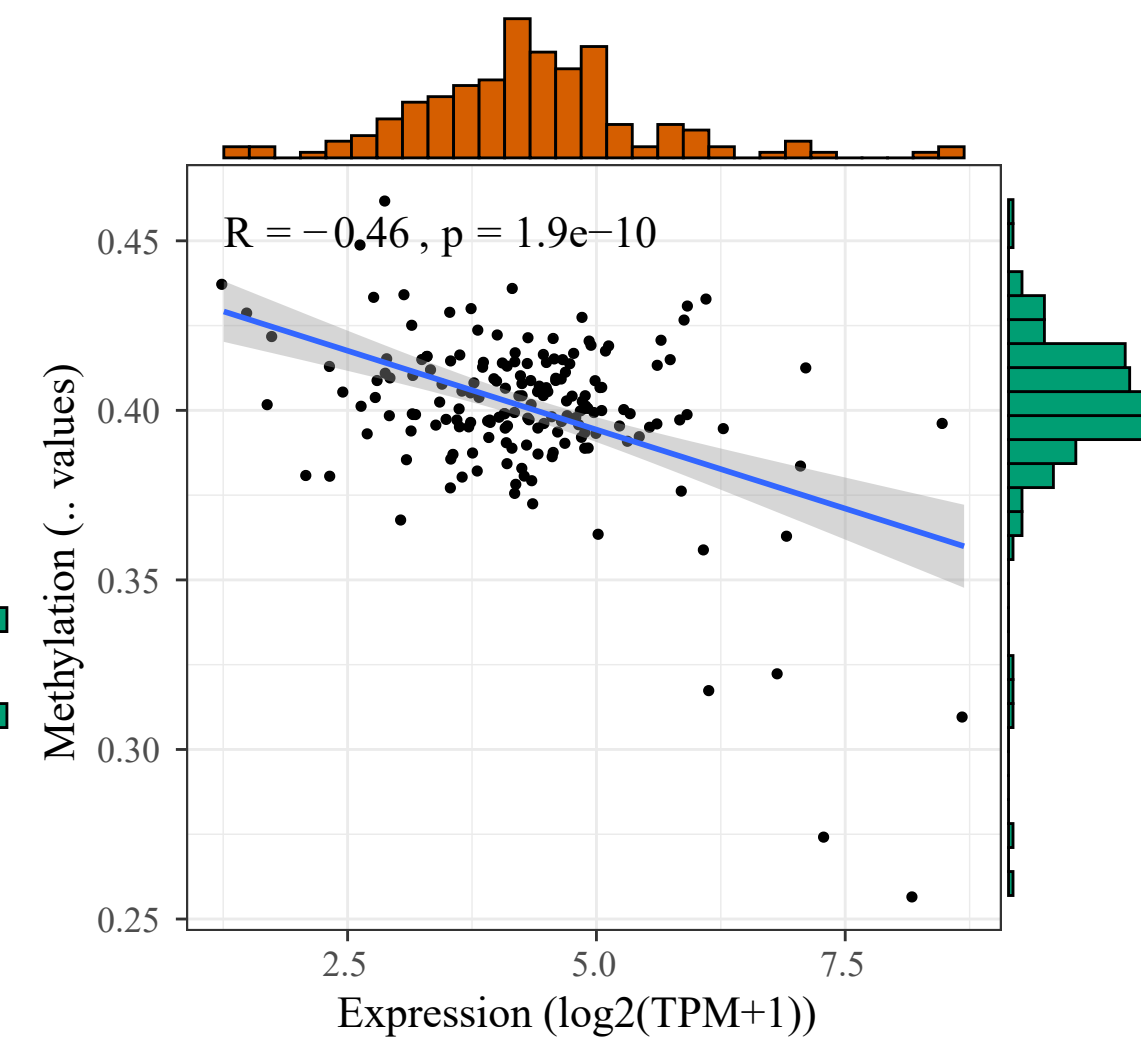

CEP55

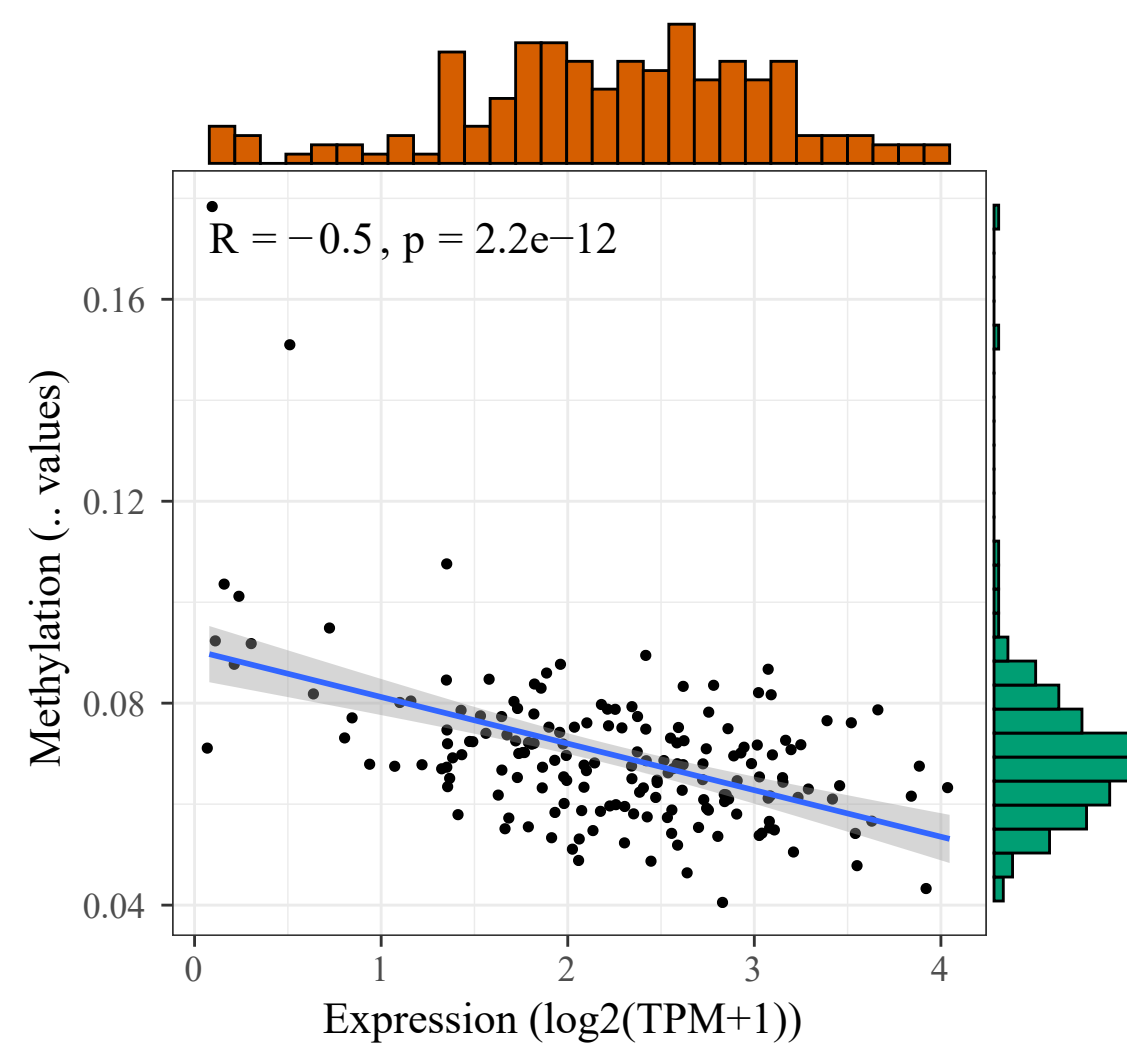

GJB4

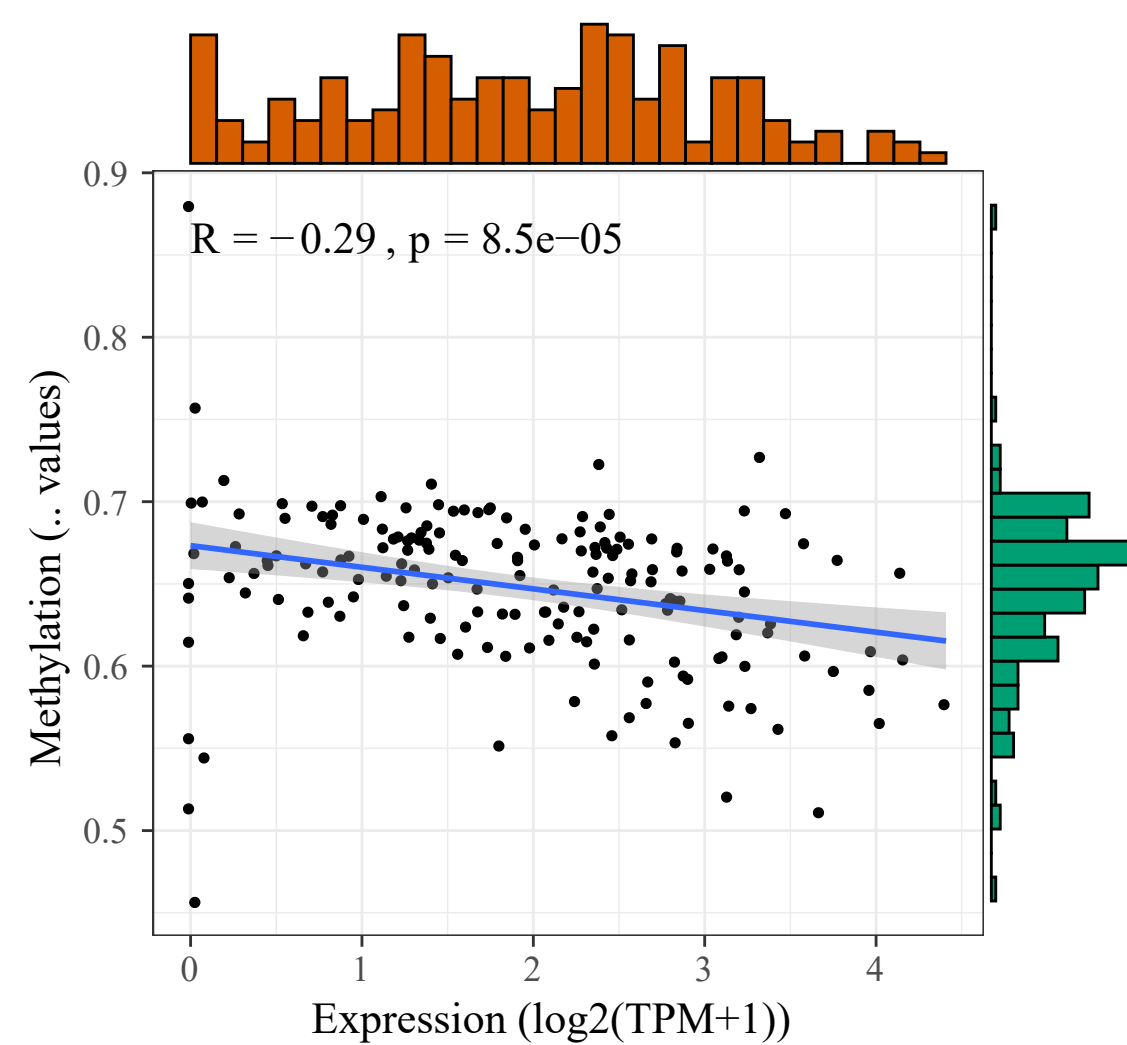

COL17A1

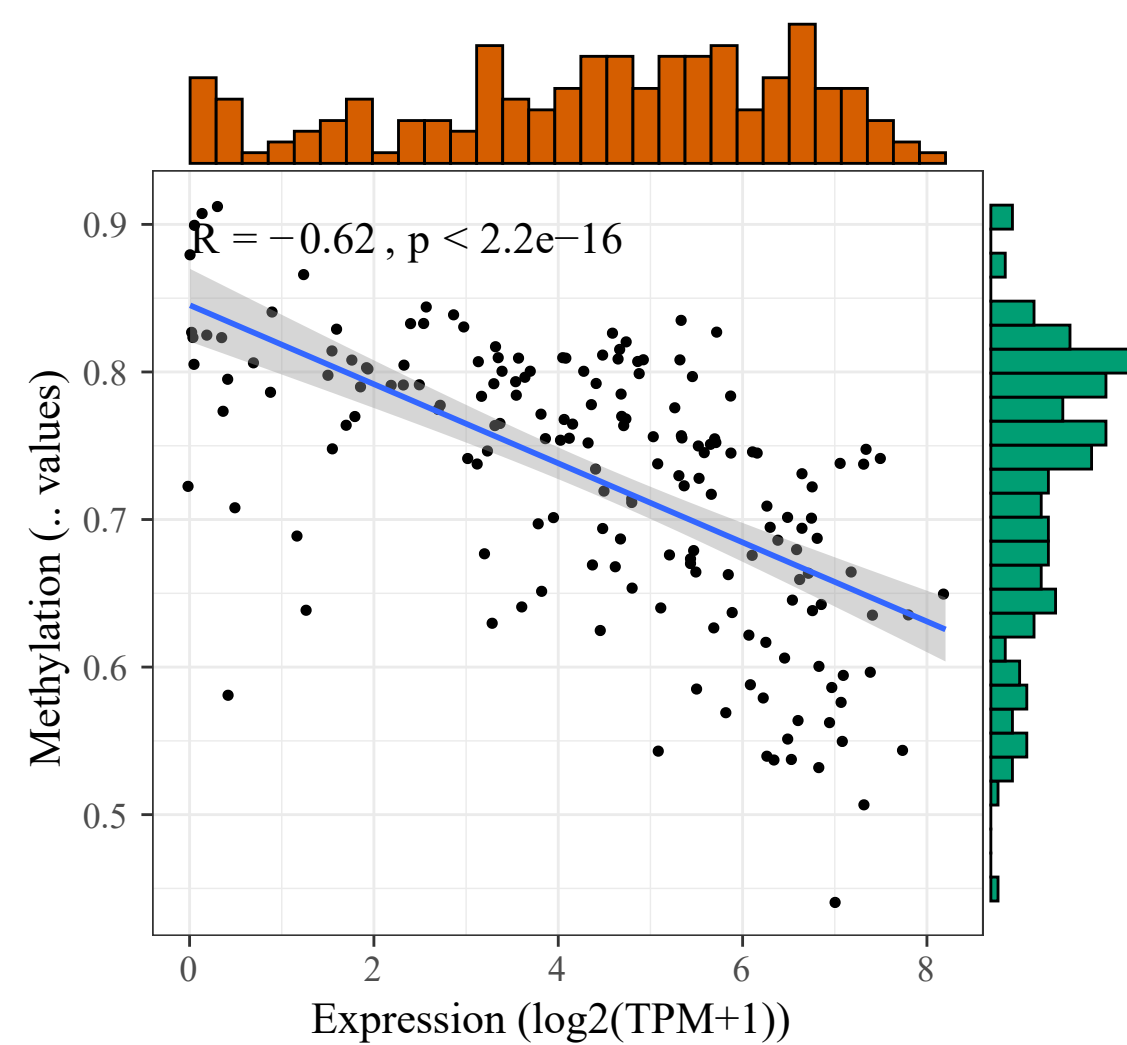

Supplement: Supplementary file 1 [file Image5.pdf]

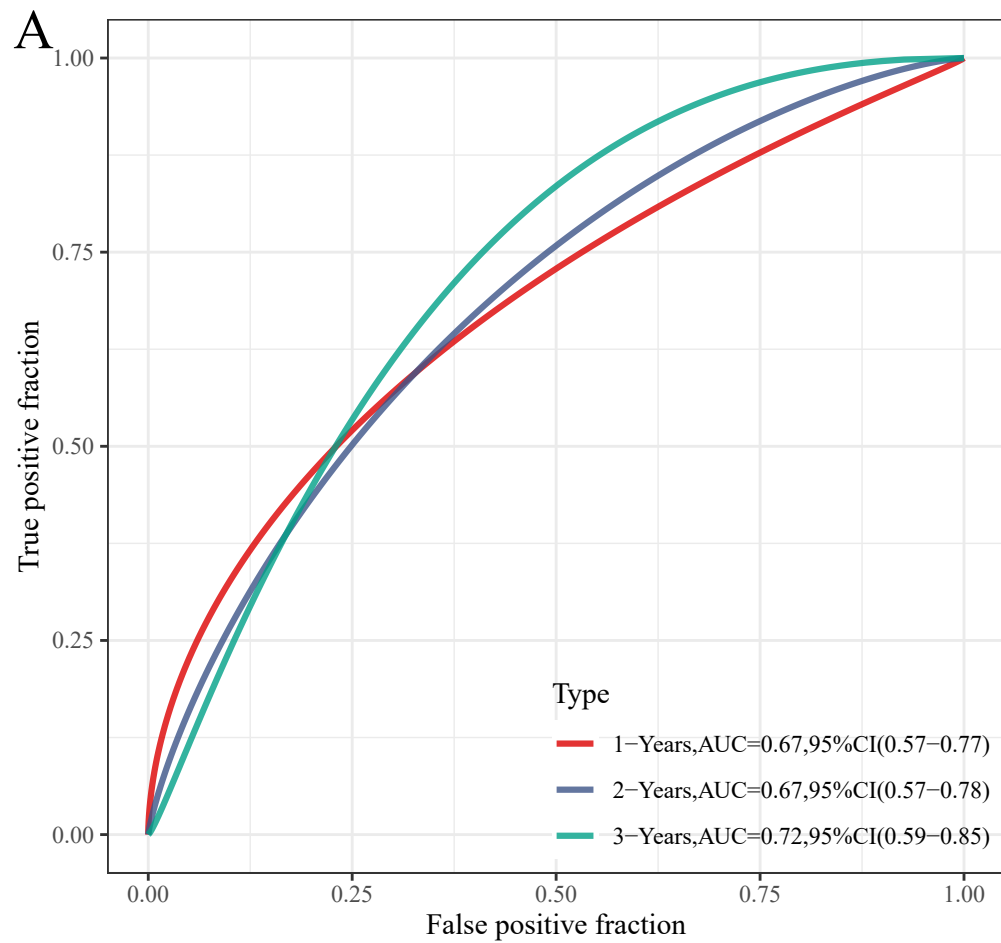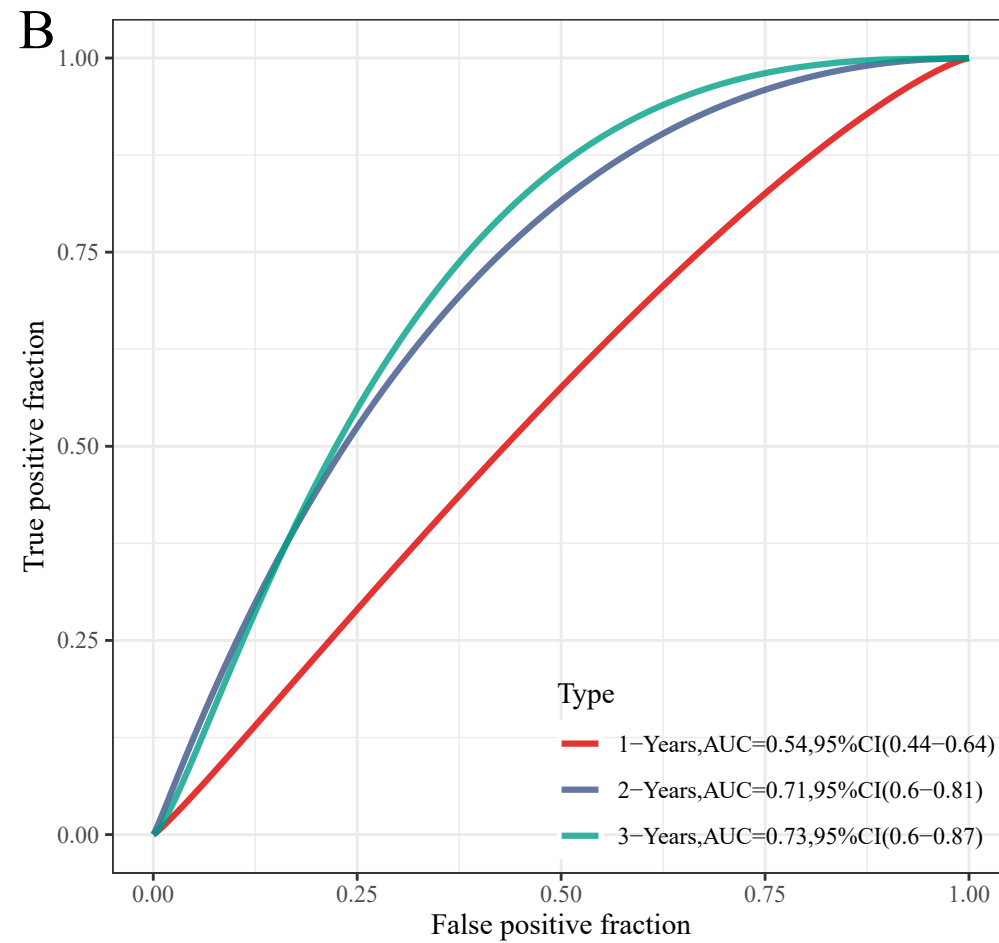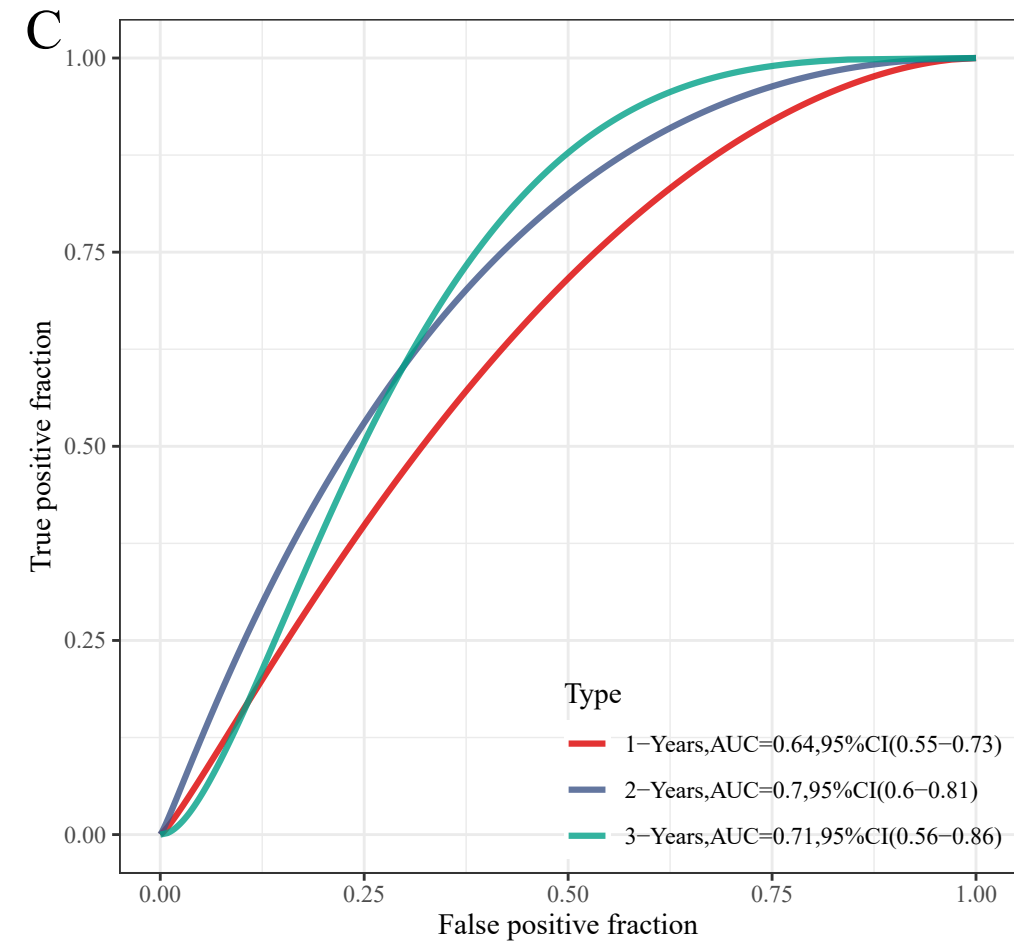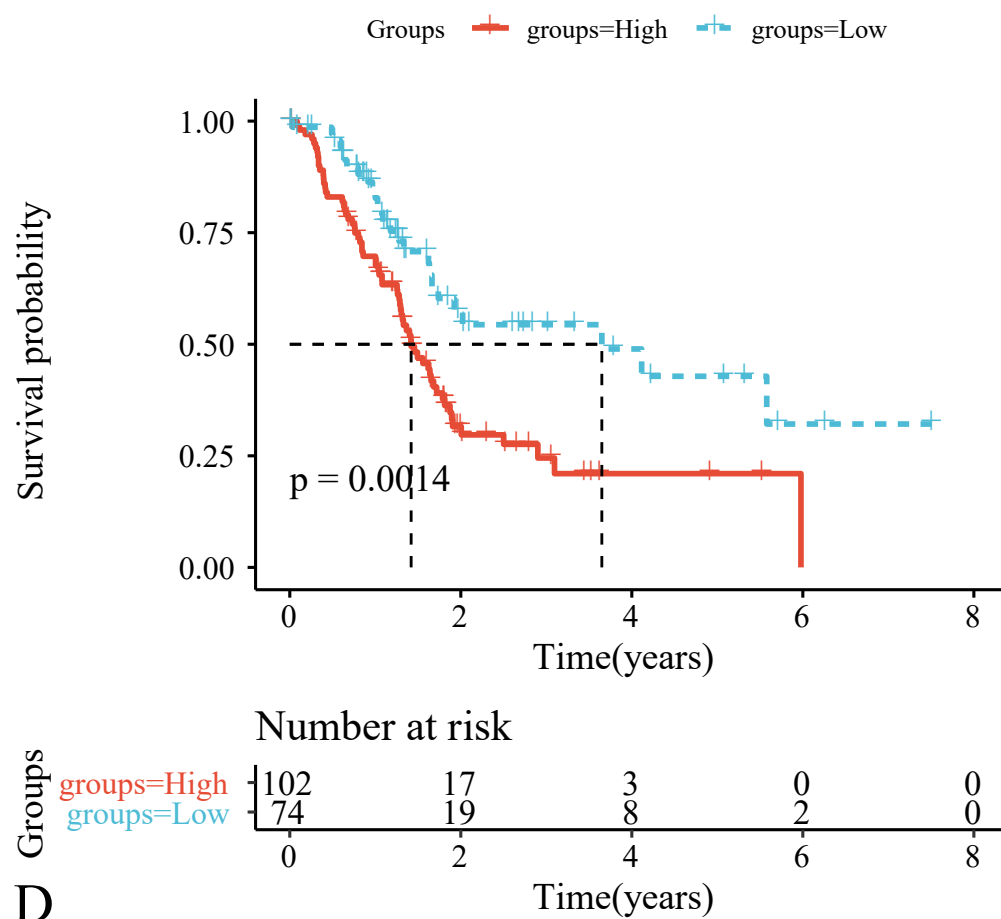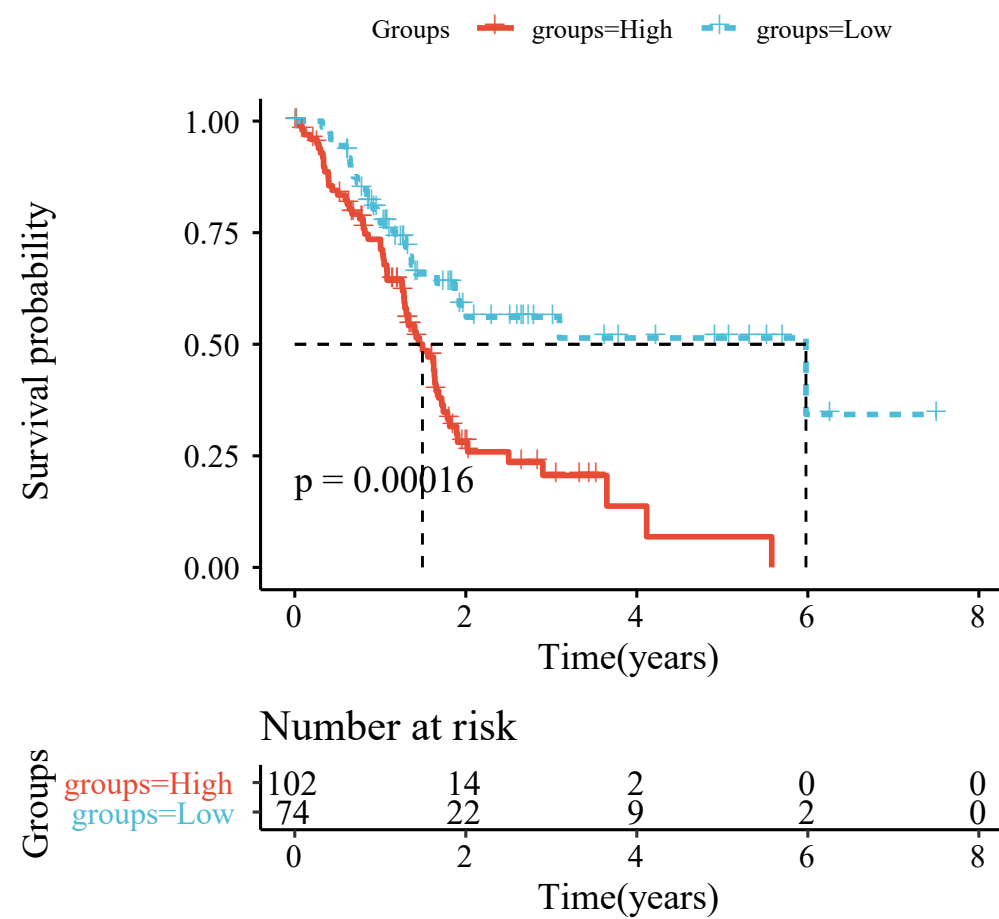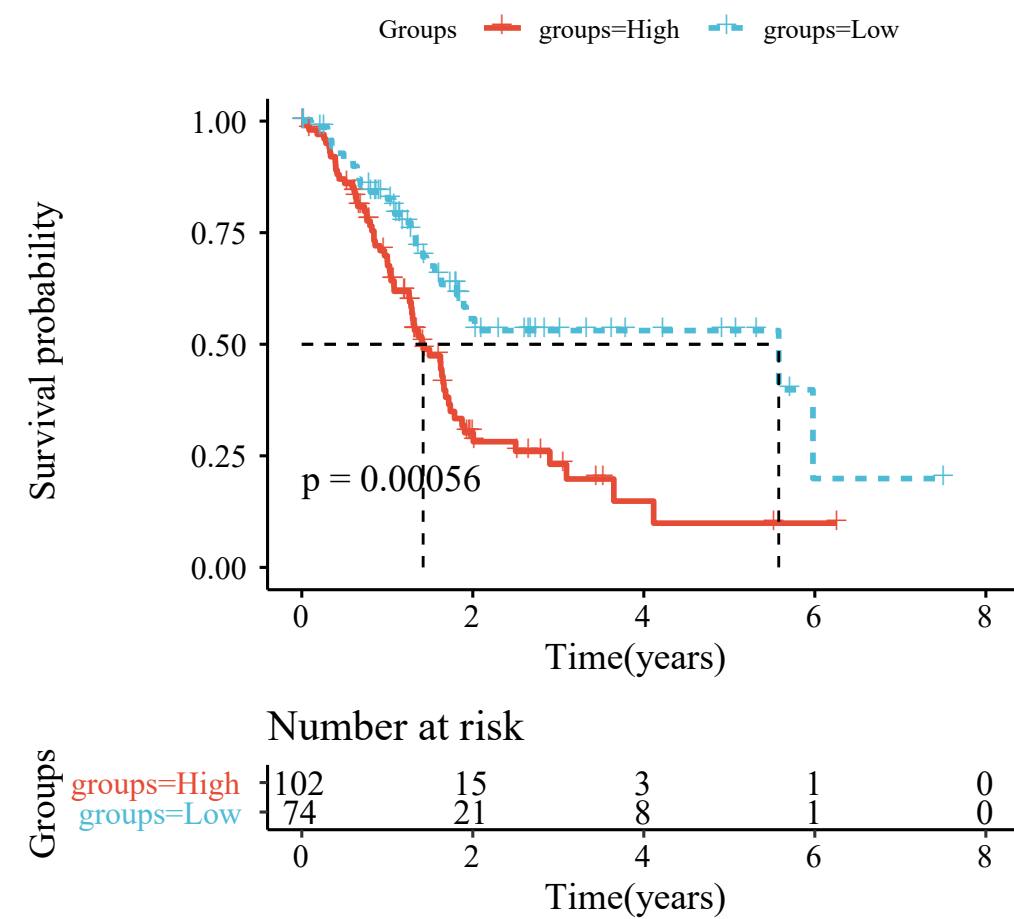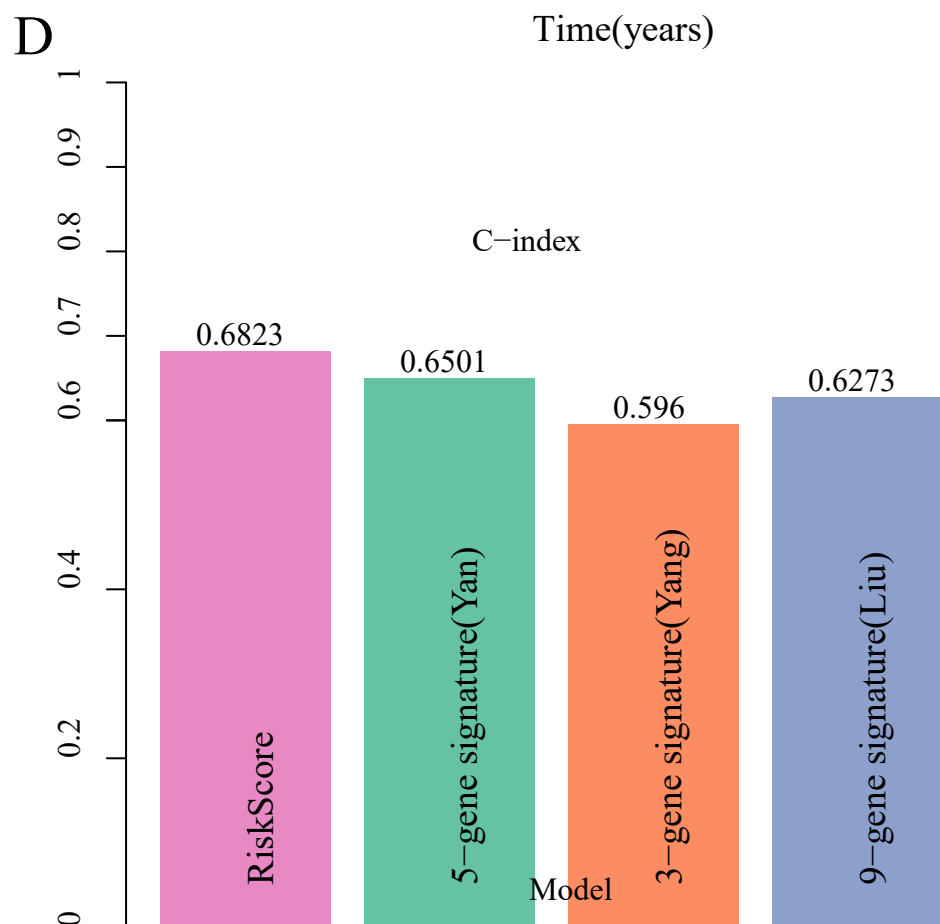

Supplement: Supplementary file 3 [file Image6.pdf]

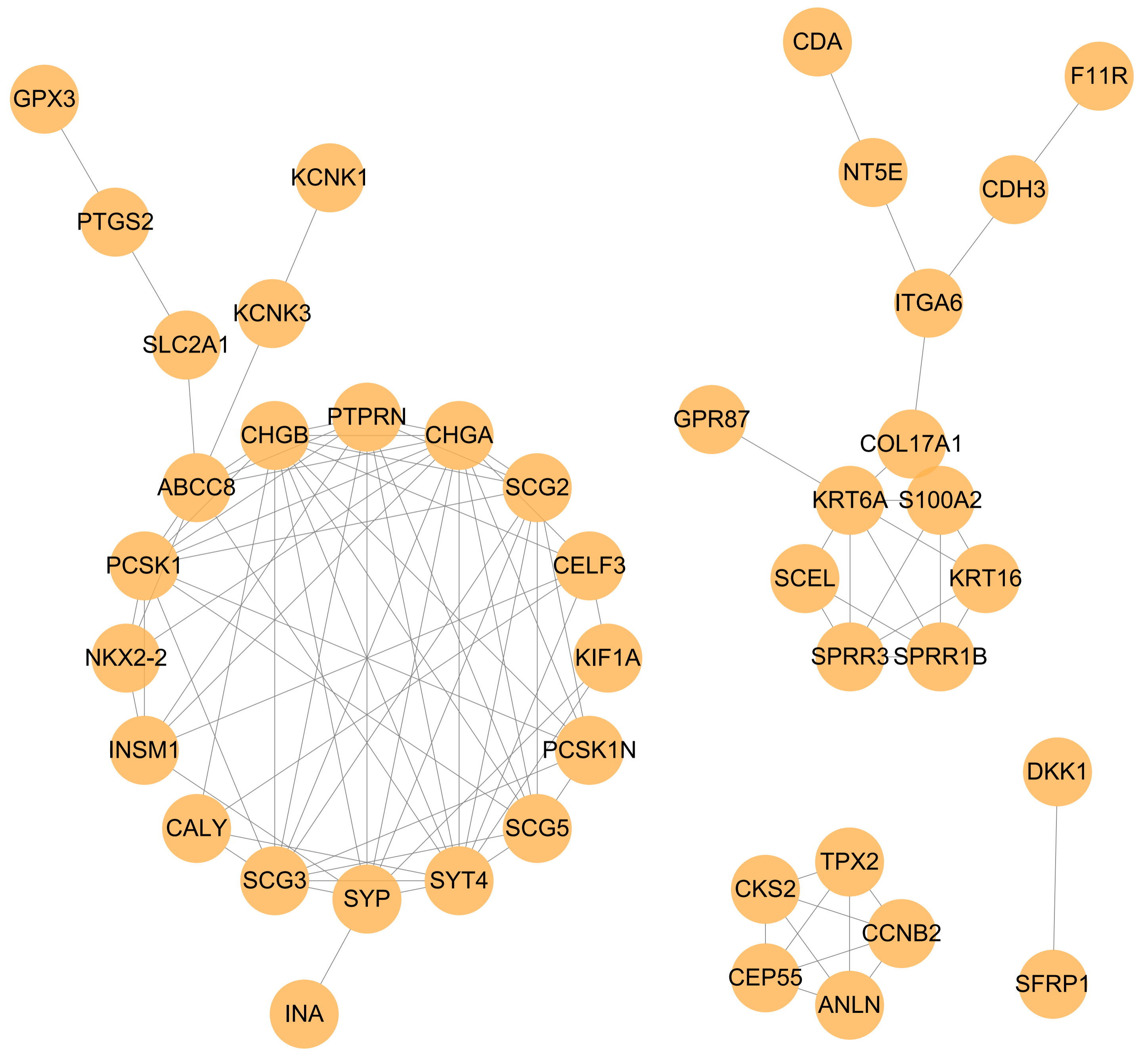

Supplement: Supplementary file 5 [file Image2.pdf]

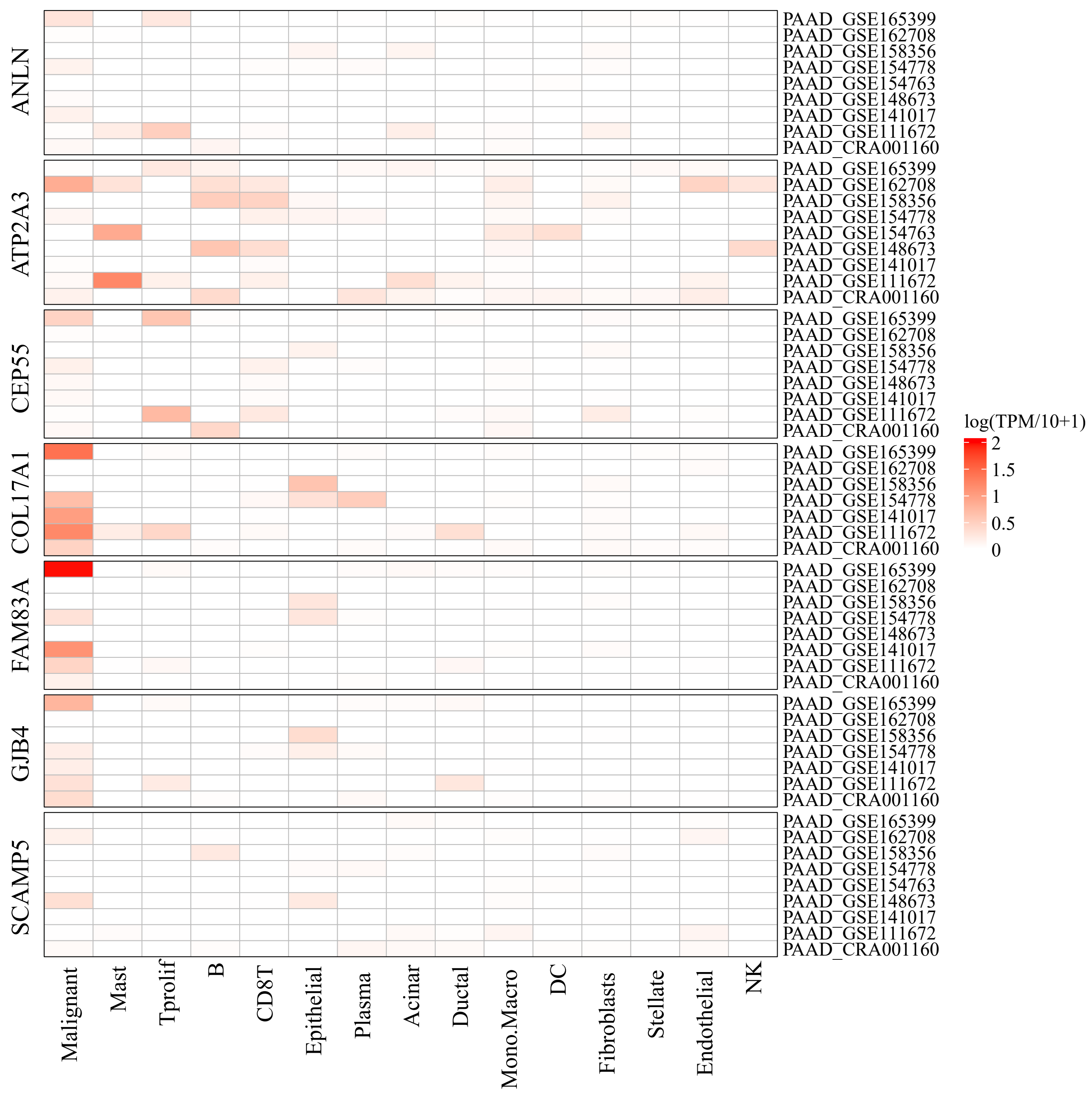

Supplement: Supplementary file 6 [file Image3.pdf]

**A**

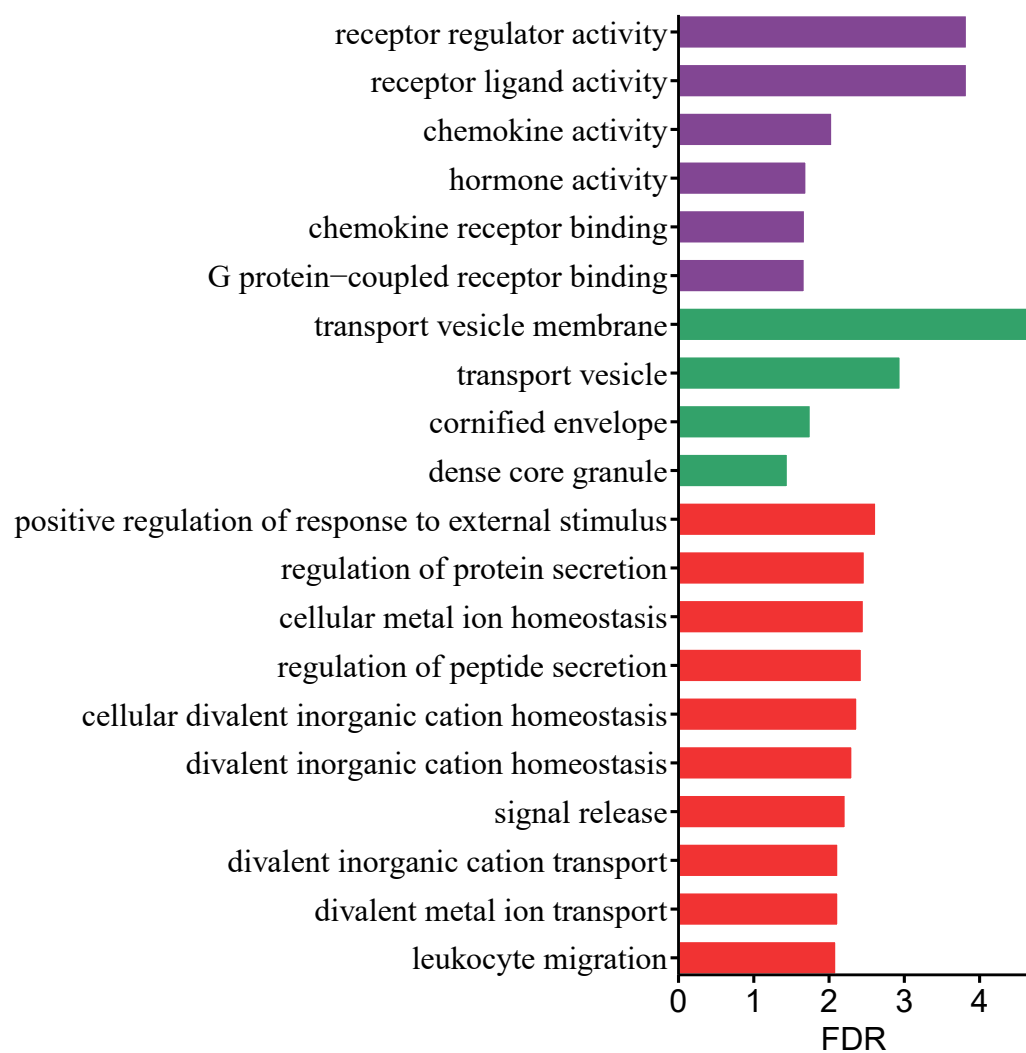

**B**

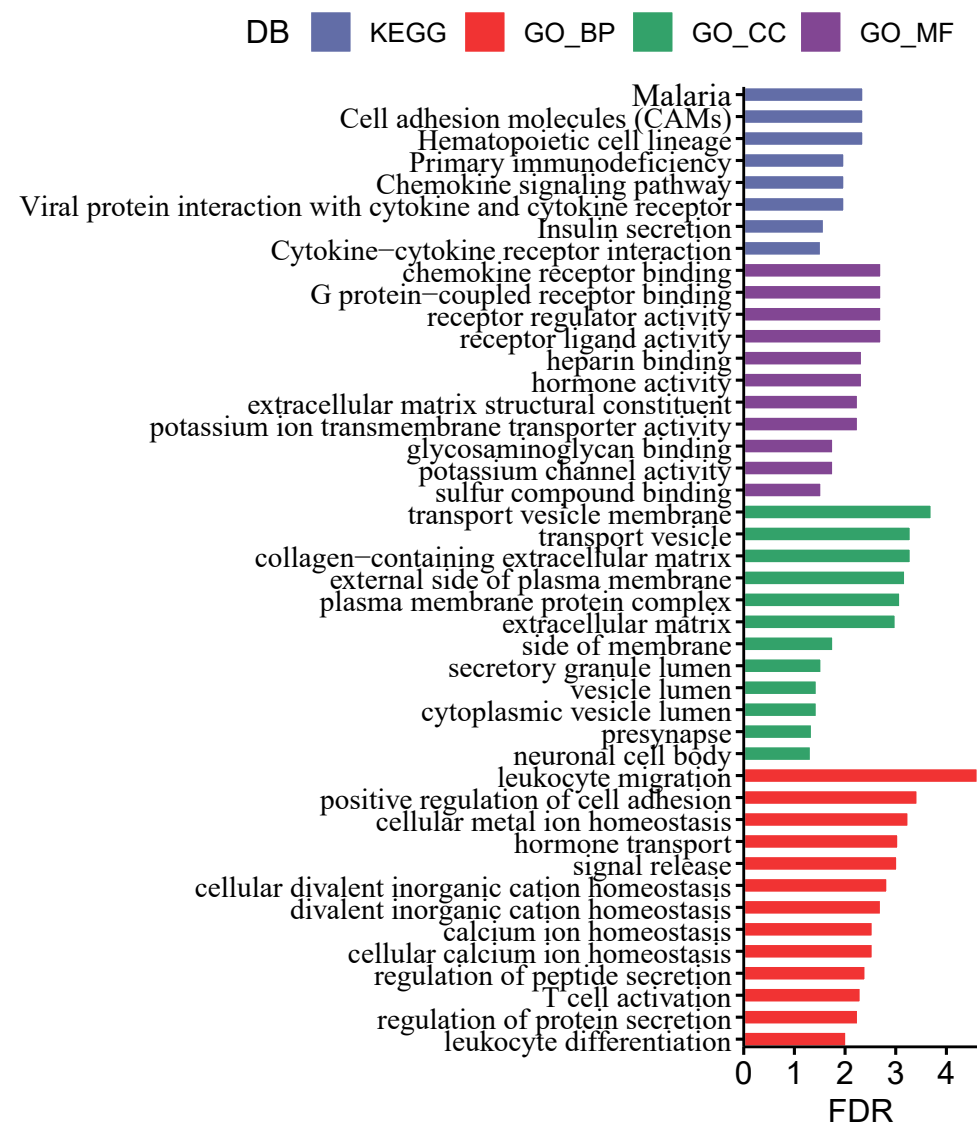

**C**

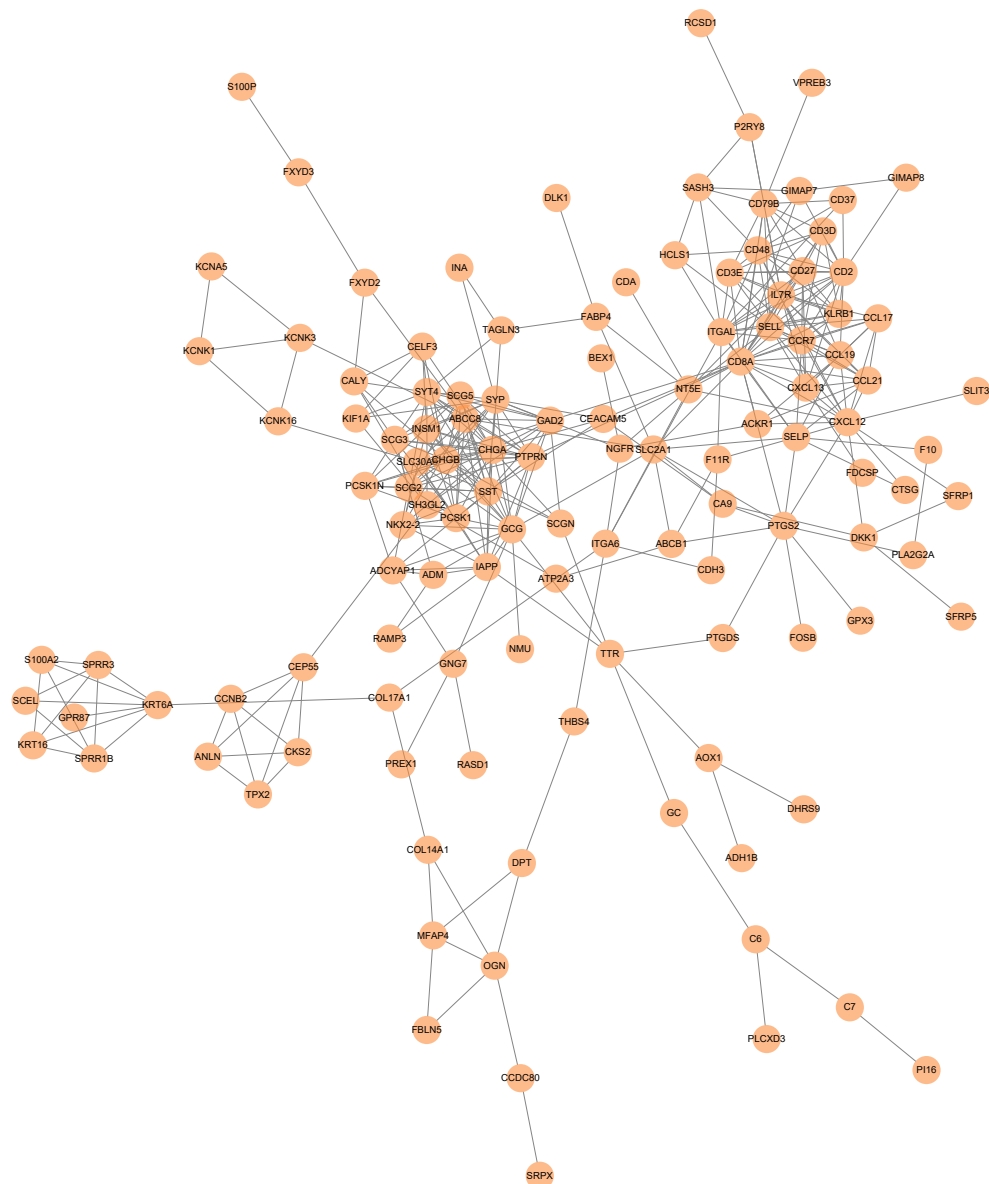

**D**

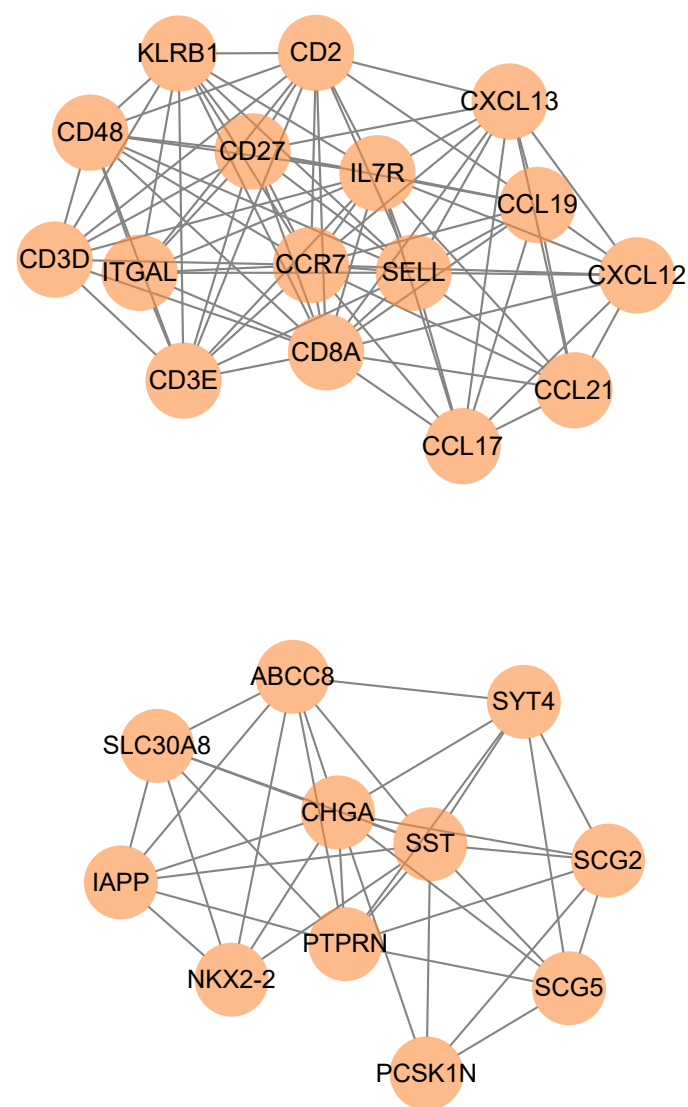

Supplement: Supplementary file 8 [file Image1.pdf]
