# Supplementary material for: Establishment of a 7-gene prognostic signature based on oxidative stress genes for predicting chemotherapy resistance in pancreatic cancer
Source: Front Pharmacol. 2023 Apr 17;14:1091378. doi: 10.3389/fphar.2023.1091378 (PMC10149707; doi:10.3389/fphar.2023.1091378)
Supplement: Supplementary file 2 [file Presentation1.docx]

**Supplementary material description**

**Supplementary Figure 1.** Pathway characteristics of different subtypes. (A) Results of GO and KEGG functional enrichment analysis of differentially expressed genes in "C1" subtype; (B) Results of GO and KEGG functional enrichment analysis of differentially expressed genes in "C3" subtype; (C) PPI network of differentially expressed genes; (D) Key clusters identified by MCODE plug-in in PPI network of differentially expressed genes; * P < 0.05; ** P < 0.01; *** P < 0.001; and **** P < 0.0001.

**Supplementary Figure 2.** PPI analysis of prognosis related DEGs.

**Supplementary Figure 3.** The analysis of 7 hub genes in various cells using Single-cell division TISCH2.

**Supplementary Figure 4.** Association between RiskScore subgroups and clinicopathological characteristics. (A) Differences of RiskScore between clinicopathological subgroups in the TCGA-PAAD cohort; (B) Differences between molecular subtypes and RiskScore subgroups in the TCGA-LUAD cohort; (C) Differences of RiskScore between clinicopathological subgroups in the PACA-AU cohort; (D) Differences between molecular subtypes and RiskScore subgroups in the PACA-AU cohort; (E) KM curves between RiskScore-high and –low groups in different clinicopathological subgroups in the TCGA-PAAD cohort; * P < 0.05; ** P < 0.01; *** P < 0.001; and **** P < 0.0001.

**Supplementary Figure 5.** The association of 7 genes expressions and methylation.

**Supplementary Figure 6.** The comparison of our model with published 3 models. A-C: the ROC and KM analysis of 3 published models. D: the comparison of C-index between our model and published 3 models.
