# Supplementary material for: Establishment of a 7-gene prognostic signature based on oxidative stress genes for predicting chemotherapy resistance in pancreatic cancer
Source: Front Pharmacol. 2023 Apr 17;14:1091378. doi: 10.3389/fphar.2023.1091378 (PMC10149707; doi:10.3389/fphar.2023.1091378)
Supplement: Supplementary file 7 [file DataSheet1.docx]

For the data analyzed in this study please see:

https://www.jianguoyun.com/p/DaQqHEQQtICMCxjf4uEEIAA
