# Supplementary material for: Establishment of a 7-gene prognostic signature based on oxidative stress genes for predicting chemotherapy resistance in pancreatic cancer
Source: Front Pharmacol. 2023 Apr 17;14:1091378. doi: 10.3389/fphar.2023.1091378 (PMC10149707; doi:10.3389/fphar.2023.1091378)
Supplement: Supplementary file 4 [file Image4.pdf]

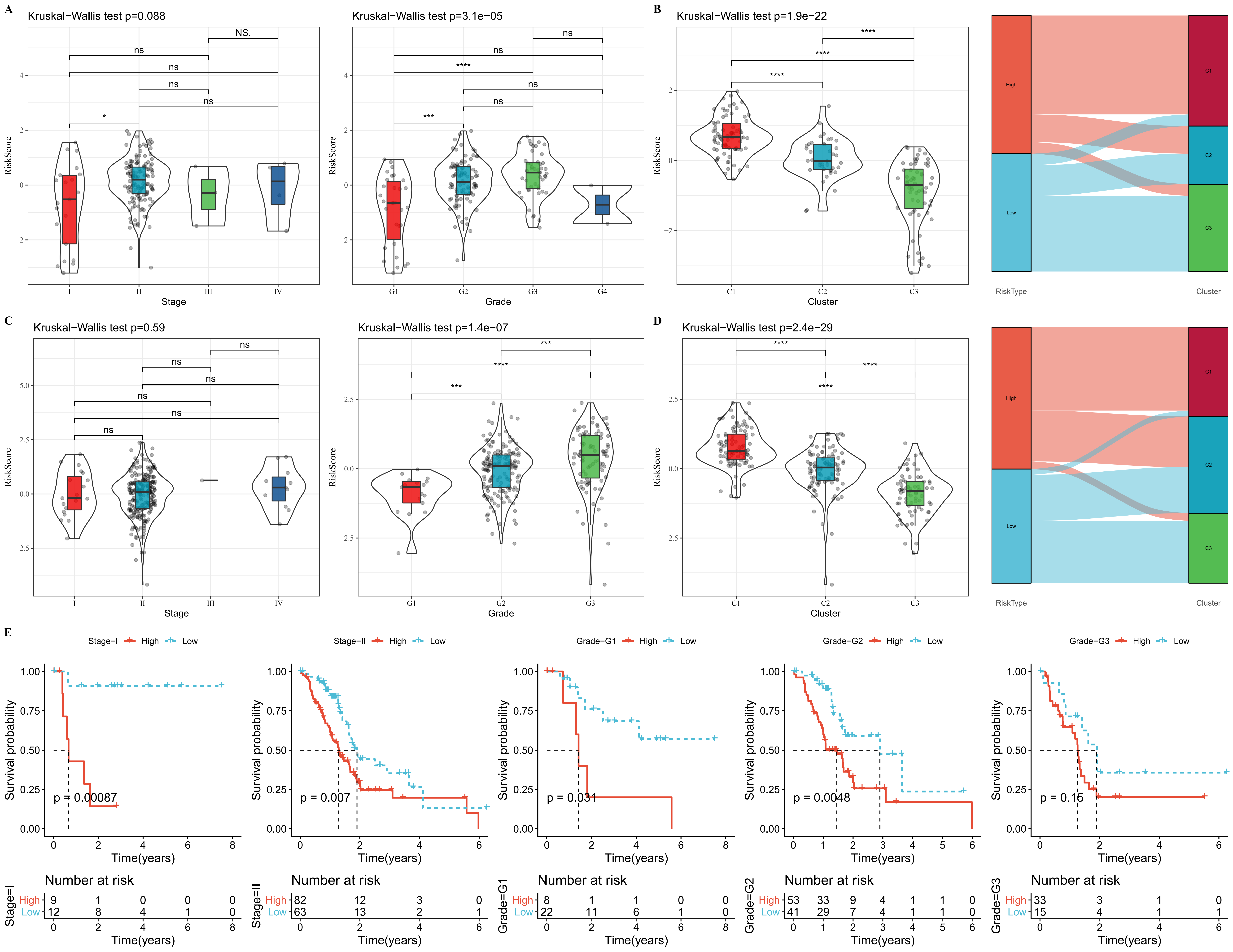
A
Kruskal−Wallis test p=0.088
NS.
ns
ns
ns
ns
*
RiskScore
I
II
III
IV
Stage
Kruskal−Wallis test p=3.1e−05
ns
ns
****
ns
ns
***
RiskScore
G1
G2
G3
G4
Grade
B
Kruskal−Wallis test p=1.9e−22
****
****
****
RiskScore
C1
C2
C3
Cluster
High
Low
C1
C2
C3
RiskType
Cluster
C
Kruskal−Wallis test p=0.59
ns
ns
ns
ns
ns
ns
RiskScore
I
II
III
IV
Stage
Kruskal−Wallis test p=1.4e−07
***
****
***
RiskScore
G1
G2
G3
Grade
D
Kruskal−Wallis test p=2.4e−29
****
****
****
RiskScore
C1
C2
C3
Cluster
High
Low
C1
C2
C3
RiskType
Cluster
E
Stage=I High Low
Survival probability
p = 0.00087
Time(years)
Number at risk
Stage=I
High 9 1 0 0 0
Low 12 8 4 1 0
Time(years)
Stage=II High Low
Survival probability
p = 0.007
Time(years)
Number at risk
Stage=II
High 82 12 3 0
Low 63 13 2 1
Time(years)
Grade=G1 High Low
Survival probability
p = 0.031
Time(years)
Number at risk
Grade=G1
High 8 1 1 0 0
Low 22 11 6 1 0
Time(years)
Grade=G2 High Low
Survival probability
p = 0.0048
Time(years)
Number at risk
Grade=G2
High 53 33 9 4 1 1 0
Low 41 29 7 4 1 1 0
Time(years)
Grade=G3 High Low
Survival probability
p = 0.16
Time(years)
Number at risk
Grade=G3
High 33 3 1 0
Low 15 4 1 1
Time(years)
